# Bifurcation of *Arabidopsis* NLR Immune Signaling via Ca^2+^-Dependent Protein Kinases

**DOI:** 10.1371/journal.ppat.1003127

**Published:** 2013-01-31

**Authors:** Xiquan Gao, Xin Chen, Wenwei Lin, Sixue Chen, Dongping Lu, Yajie Niu, Lei Li, Cheng Cheng, Matthew McCormack, Jen Sheen, Libo Shan, Ping He

**Affiliations:** 1 Department of Biochemistry and Biophysics, and Institute for Plant Genomics and Biotechnology, Texas A&M University, College Station, Texas, United States of America; 2 Department of Plant Pathology and Microbiology, and Institute for Plant Genomics and Biotechnology, Texas A&M University, College Station, Texas, United States of America; 3 Department of Biology, Genetics Institute, Plant Molecular and Cellular Biology Program, Interdisciplinary Center for Biotechnology Research, University of Florida, Gainesville, Florida, United States of America; 4 Department of Genetics, Harvard Medical School, and Department of Molecular Biology and Center for Computational and Integrative Biology, Massachusetts General Hospital, Boston, Massachusetts, United States of America; Michigan State University, United States of America

## Abstract

Nucleotide-binding domain leucine-rich repeat (NLR) protein complexes sense infections and trigger robust immune responses in plants and humans. Activation of plant NLR resistance (R) proteins by pathogen effectors launches convergent immune responses, including programmed cell death (PCD), reactive oxygen species (ROS) production and transcriptional reprogramming with elusive mechanisms. Functional genomic and biochemical genetic screens identified six closely related *Arabidopsis* Ca^2+^-dependent protein kinases (CPKs) in mediating bifurcate immune responses activated by NLR proteins, RPS2 and RPM1. The dynamics of differential CPK1/2 activation by pathogen effectors controls the onset of cell death. Sustained CPK4/5/6/11 activation directly phosphorylates a specific subgroup of WRKY transcription factors, WRKY8/28/48, to synergistically regulate transcriptional reprogramming crucial for NLR-dependent restriction of pathogen growth, whereas CPK1/2/4/11 phosphorylate plasma membrane-resident NADPH oxidases for ROS production. Our studies delineate bifurcation of complex signaling mechanisms downstream of NLR immune sensors mediated by the myriad action of CPKs with distinct substrate specificity and subcellular dynamics.

## Author Summary

Distinguishing self from non-self is the fundamental principle of immunity. Nucleotide-binding leucine-rich repeat (NLR) proteins were first identified in plants as disease resistance proteins and were recently found to play essential roles in mammalian innate immunity and inflammation. NLR protein complexes sense intracellular pathogenic effectors in plants and microbial patterns and danger signals in humans, but the signaling mechanisms upon NLR activation remain elusive. Using the *Arabidopsis-Pseudomonas* interaction as a model system, we discovered the molecular link between NLR immune sensors and the convergent immune responses triggered by distinct pathogen effectors. Integrated functional genomic and biochemical genetic screens identified six closely related Ca^2+^-dependent protein kinases (CPKs) that orchestrate bifurcate NLR immune signaling via distinct substrate specificity and subcellular dynamics. The CPK1/2 regulate the onset of programmed cell death; CPK4/5/6/11 phosphorylate specific WRKY transcription factors to regulate immune gene expression crucial for NLR-dependent restriction of pathogen growth, whereas CPK1/2/4/11 phosphorylate NADPH oxidases for the production of reactive oxygen species. Our studies decode the complex signaling mechanisms via the myriad action of CPKs downstream of NLR immune sensors.

## Introduction

The first line of nonself recognition and immune responses in multicellular organisms is triggered by conserved pathogen- or microbe-associated molecular patterns (PAMPs/MAMPs) through pattern recognition receptors (PRRs). MAMPs, such as bacterial flagellin and peptidoglycan (PGN) or fungal chitin, are perceived by cell-surface receptors to mount PAMP/MAMP-triggered immunity (PTI) for broad-spectrum microbial resistance in plants [1], [2]. Successful pathogens acquired virulence effectors to suppress PTI. To confine or eliminate pathogens, plants further evolved polymorphic R proteins to directly or indirectly recognize effectors and initiate effector-trigger immunity (ETI) accompanied with localized PCD and systemic defense signaling [3], [4], [5], [6], [7]. The most common R proteins are intracellular immune sensors with the nucleotide-binding domain (NB) and leucine-rich repeat (LRR), a structural feature shared by mammalian NOD-like receptors that perceive intracellular MAMPs and danger signals to initiate inflammation and immunity [6], [8], [9], [10], [11], [12]. Whether and how distinct intracellular and cell-surface immune sensors trigger overlapping or/and differential primary immune signaling responses are still largely open questions.

In *Arabidopsis thaliana*, NLR protein RPS2 initiates resistance upon recognition of *Pseudomonas syringae* effector AvrRpt2, whereas RPM1 recognizes two sequence-unrelated effectors, AvrRpm1 and AvrB. With a few exceptions, NLR proteins do not interact directly with pathogen effectors, but instead monitor perturbation of host proteins by pathogen effectors to mount defense responses [3], [4], [5], [6], [7], [8], [9], [10]. For instance, AvrRpt2 degrades *Arabidopsis* RIN4 protein to activate RPS2 signaling, whereas AvrRpm1 and AvrB induce RIN4 phosphorylation via host kinases to initiate RPM1 signaling [13], [14], [15], [16]. Although several plant NLR proteins, such as barley MLA10 [17], tobacco N [18] and *Arabidopsis* RPS4 [19], [20], require effector-induced nuclear translocation for immune signaling, RPS2 and RPM1 are anchored to the plasma membrane to elicit immune responses [15], [21]. Potato Rx protein requires both nuclear and cytoplasmic localizations for full immunity [22], [23]. Apparently, different NLR proteins deploy distinct mechanisms in multiple subcellular compartments to activate complex downstream signaling. The molecular link between the activated NLR proteins and the diverse downstream signaling events that lead to PCD activation, ROS production and transcriptional reprogramming has remained elusive.

Ca^2+^ is an essential and conserved second messenger in nearly every aspect of cellular signaling programs. Ca^2+^ influx is a prerequisite for PCD triggered by AvrRpm1/AvrB-RPM1 and AvrRpt2-RPS2 interactions [24], [25], [26]. How the Ca^2+^ signal is sensed and transduced upon NLR protein activation has remained obscure. There are three major types of Ca^2+^ sensors in plants, including calmodulin (CAM), calcineurin B-like proteins and calcium-dependent protein kinases (CPKs) [27], [28], [29]. It has been shown that *Arabidopsis* CAM-like protein CML24 is required for nitric oxide (NO) production and AvrRpt2-mediated PCD [26]. CPKs have been identified ubiquitously throughout the plant kingdom and share a protein kinase domain with high sequence homology to the mammalian multifunctional CAM-dependent protein kinases, suggesting their dual function as Ca^2+^ sensors and signal transducers [27], [29]. Tobacco CPKs play essential roles in PCD induced by Avr9-Cf9 interaction, in which Cf9 encodes a cell-surface receptor with an N-terminal LRR domain [30], [31]. Potato StCPK4 and StCPK5 directly phosphorylate and activate NADPH oxidase RBOHB (Respiratory Burst Oxidase Homologue B) [32]. There are 34 CPKs in *Arabidopsis* genome, which can be classified into four groups (I–IV) based on sequence similarity [27]. Recently, four *Arabidopsis* CPKs (CPK4/5/6/11) have been identified to play important roles, together with the MAPK cascades, in relaying primary MAMP immune signaling [33]. Distinct from the rapid and transient increase of cytosolic Ca^2+^ concentration induced by MAMPs [34], [35], [36], inoculation with bacteria carrying *avrRpm1*, *avrB* or *avrRpt2* triggered a much prolonged and sustained increase of cytosolic Ca^2+^ concentration accompanied with PCD in *Arabidopsis* leaves [25], [26]. It remains enigmatic how the distinct calcium signatures are sensed and relayed for differential and overlapping immune responses in ETI and PTI signaling.

In the present study, we have identified six *Arabidopsis* CPKs in sensing and transducing Ca^2+^ signatures dynamically activated by RPS2 and RPM1 upon AvrRpt2 and AvrRpm1/AvrB elicitation, respectively. The specificity and redundancy of individual CPKs in NLR signaling events, including CPK4/5/6/11 in orchestrating immune gene expression, CPK1/2/4/11 in ROS production, and CPK1/2/5/6/ in PCD, were revealed by integrative biochemical, cellular, functional genomic and genetic analyses. Apparently, specific CPKs are engaged in diverse immune responses via phosphorylation and activation of different substrates in distinct subcellular compartments. Functional genomic screens identified a specific subgroup of WRKY transcription factors that act synergistically with CPKs in primary NLR signaling. Sustained activation of CPK4/5/6/11 phosphorylates WRKY8/28/48 for transcriptional reprogramming of immune genes, whereas CPK1/2/4/11 phosphorylate NADPH oxidases for ROS production and contribute to PCD. Our results reveal bifurcate NLR signaling mechanisms through specific, overlapping and prolonged actions of CPKs in concert with distinct substrates in multiple subcellular compartments.

## Results

### PCD and immune gene activation triggered by bacterial effectors

**Figure 1:**
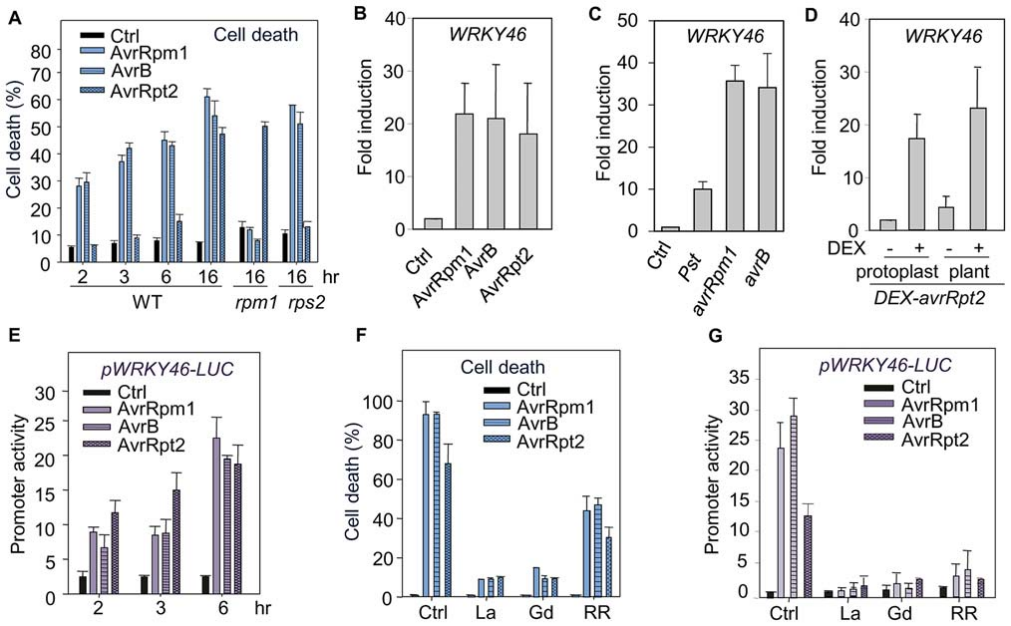
The requirement of Ca^2+^ signaling in ETI. (**A**) AvrRpm1-, AvrB- and AvrRpt2-induced cell death was detected by Evan's blue staining at different time points after transfection in WT, *rpm1* or *rps2* protoplasts. Ctrl is a control vector. Data are shown as mean ± SD. (**B**) AvrRpm1, AvrB and AvrRpt2 activated endogenous *WRKY46* expression in protoplasts. The transfected protoplasts were collected 6 hpt for real-time RT-PCR analysis. The expression of *WRKY46* was normalized to the expression of *UBQ10*. The data are shown as the mean ± SE from three independent biological replicates. (**C**) Induction of *WRKY46* by *Pst avrRpm1* and *avrB* infection in plants. Plant leaves were hand-inoculated with control or bacteria at 1×10^7^ cfu/ml. The samples were collected 6 hpi for real-time RT-PCR analysis. The expression of *WRKY46* was normalized to the expression of *UBQ10*. The data are shown as the mean ± SE from three independent biological replicates. (**D**) Induction of *WRKY46* in dexamethasone (DEX)-inducible *avrRpt2* transgenic plants and protoplasts. The *WRKY46* expression was detected 6 hr after DEX treatment with real-time RT-PCR analysis. The expression of *WRKY46* was normalized to the expression of *UBQ10*. The data are shown as the mean ± SE from three independent biological replicates. (**E**) AvrRpm1, AvrB and AvrRpt2 activated *WRKY46* promoter in protoplasts. The p*WRKY46-LUC* was co-transfected with *avrRpm1*, *avrB*, or *avrRpt2*, or a vector control in protoplasts and samples were collected at indicated time points. The *UBQ-GUS* was included as an internal transfection control. The relative luciferase activity was normalized with GUS activity. (**F**) AvrRpm1, AvrB and AvrRpt2-induced cell death was suppressed by calcium inhibitors in *Arabidopsis* protoplasts. The *avrRpm1*, *avrB*, or *avrRpt2* was co-transfected with *UBQ-GUS* and incubated with 1 mM LaCl_3_, 1 mM GdCl_3_ or 10 µM RR. The samples were collected 16 hpt, and the cell death ratio was presented as the percentage of GUS activity repression in effector-transfected cells compared to control-transfected cells. (**G**) Effector-induced *WRKY46* promoter activity was suppressed by calcium inhibitors in protoplasts. The samples were collected 6 hpt. The above experiments were repeated at least four times with similar results.

To elucidate early signaling events in plant ETI, we have deployed an *Arabidopsis* mesophyll protoplast system in which pathogen-encoded individual effector genes are expressed to monitor specific and temporal responses. The cell-autonomous and synchronized elicitation in a homogeneous cell population by a single pathogen effector circumvents the complex responses simultaneously activated or/and repressed by a large array of MAMPs and effectors in intact plant-pathogen interactions [37], [38]. Expression of effector gene, *avrRpm1*, *avrB* or *avrRpt2*, in protoplasts triggered distinct kinetics of PCD as detected by Evan's blue staining (Figure 1A). The PCD induced by AvrRpm1 or AvrB was observed as early as 2 hr post-transfection (hpt), whereas the PCD induced by AvrRpt2 was evident at 16 hpt, reminiscent of observations with the actual plant-pathogen interactions (Figure S1A) [39]. PCD was not detected in the corresponding NLR mutants *rpm1* and *rps2* (Figure 1A). Effector-induced PCD was accompanied by enhanced nuclear fragmentation visualized by fluorescent YO-PRO-1 iodide staining (Figure S1B), consistent with a previous report based on direct effector protein delivery [38].

We performed a genome-wide transcriptome analysis of protoplasts expressing *avrRpm1* or *avrRpt2*, and identified *WRKY46* as an early marker gene in convergent ETI signaling (data not shown). The *WRKY46* transcript was strongly induced in protoplasts expressing *avrRpm1*, *avrB* or *avrRpt2* in an RPM1 or RPS2 dependent manner (Figure 1B and S1C). The induction of *WRKY46* by effectors was further confirmed with plants infected by *P. syringae* DC3000 (*Pst*) carrying *avrRpm1* or *avrB* (Figure 1C) and in dexamethasone-inducible *avrRpt2* transgenic plants (Figure 1D and S1D). Similar to the endogenous gene, the promoter of *WRKY46* fused to a firefly luciferase reporter gene (*LUC*) was strongly activated by AvrRpm1, AvrB or AvrRpt2 in protoplasts (Figure 1E). Notably, unlike PCD, effector-induced *WRKY46* activation was observed to follow with similar kinetics, as early as 2 hpt, suggesting distinct mechanisms governing PCD and immune gene activation.

### Differential CPK activation in ETI signaling

To elucidate the signaling mechanisms underlying PCD and gene activation triggered by different bacterial effectors, we first explored chemical inhibitors affecting various Ca^2+^ channels. Consistent with previous reports, the calcium-channel blocker, LaCl_3_, suppressed effector-mediated PCD in *Arabidopsis* leaves inoculated with *Pst avrRpm1* or *avrRpt2* (Figure S2A) [24], [25]. Interestingly, effector-mediated PCD was also significantly diminished in the presence of ruthenium red (RR), which inhibits Ca^2+^ release from internal stores (Figure S2A). The similar effects of calcium-channel blockers were observed in protoplasts expressing AvrRpm1, AvrB or AvrRpt2 (Figure 1F), validating the responses in whole leaves and mesophyll single-cell system. These Ca^2+^ inhibitors also suppressed effector-mediated *WRKY46* promoter activation (Figure 1G). Thus, both external and internal sources of Ca^2+^ are essential in ETI signaling.

**Figure 2:**
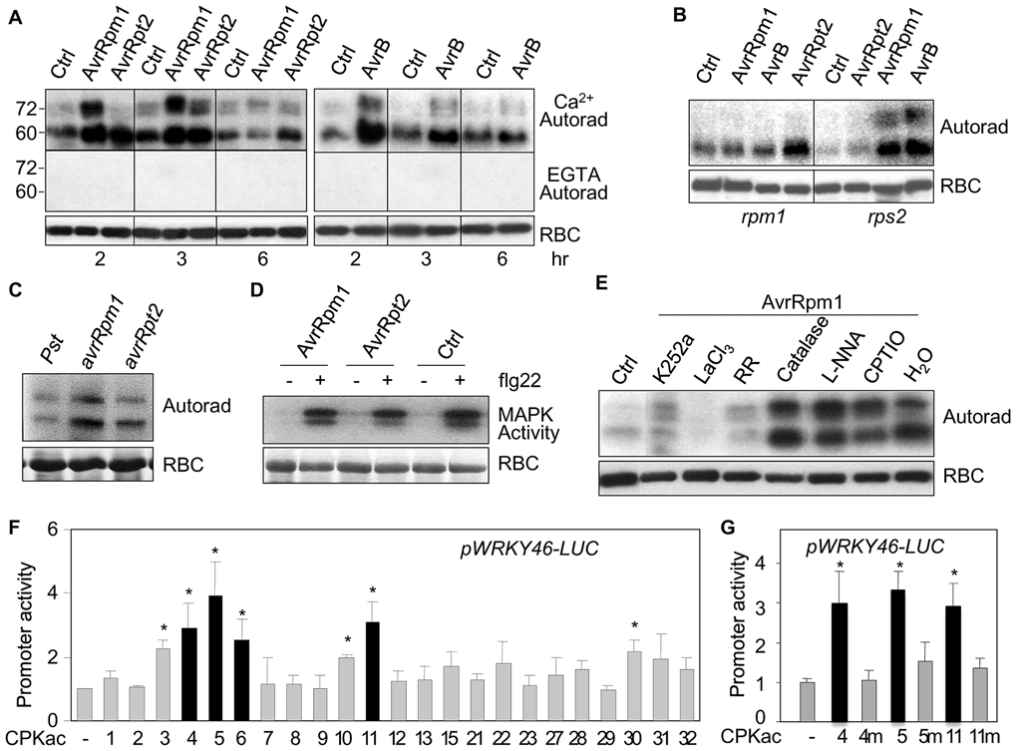
The involvement of CPKs in ETI. (**A**) Effectors activated endogenous CPKs in protoplasts. Protoplasts were collected at indicated time points after transfection with Ctrl, *avrRpm1*, *avrRpt2*, or *avrB*. The kinase activity was analyzed with an in-gel kinase assay using histone type III-S as a substrate in the presence of 0.2 mM CaCl_2_ or 2 mM EGTA. RBC (RuBisCo) is a loading control by Western blot with an α-RBC antibody. (**B**) Effector-mediated CPK activation depended on the corresponding host NLR proteins in protoplasts. The in-gel kinase assay was performed 2 hpt. (**C**) Activation of CPKs by *Pst avrRpm1* or *avrRpt2* in plants. Four-week old *Arabidopsis* plants were inoculated with *Pst*, *Pst avrRpm1* or *avrRpt2* at 1×10^8^ cfu/ml. The samples were collected 2 hpi for in-gel kinase assay with histone type III-S as a substrate. (**D**) Differential activation of MAPKs by flagellin and effectors in protoplasts. Ctrl, *avrRpm1*, or *avrRpt2*-transfected cells were incubated for 3 hr before treatment with 1 µM flg22 (22-amino-acid peptide of flagellin) for 10 min and subjected for an in-gel kinase assay using MBP as substrate. (**E**) Activation of CPKs in the presence of different chemical inhibitors in protoplasts. The concentration of inhibitors: K252a, 0.2 µM; LaCl_3_, 1 mM; RR, 10 µM; Catalase, 0.5 mg/ml; L-NNA, 100 µM; CPTIO, 100 µM. (**F**) Functional genomic screen of CPKacs in protoplasts. The *pWRKY46-LUC* was co-transfected with individual *CPKacs* to determine the activation of *WRKY46* promoter. The data are shown as the mean ± SE (n = 3) and the asterisk (*) indicates a significant difference between CPKac and control (p<0.05). (**G**) Kinase dependence of *WRKY46* promoter activation by CPKacs in protoplasts. “m” indicates the kinase-dead mutants of CPKacs. The above experiments were repeated three to four times with similar results.

To investigate the potential involvement of CPKs in ETI signaling, we developed an in-gel kinase assay using histone as a general substrate. Interestingly, different effectors activated two major groups of putative endogenous CPKs with distinct molecular masses and kinetics in a Ca^2+^ dependent manner (Figure 2A). The activation of 72-kDa CPKs by AvrRpm1 or AvrB appeared stronger and occurred earlier (2 hpt) than the corresponding responses induced by AvrRpt2 (3 hpt), whereas the activation of 60-kDa CPKs displayed similar kinetics triggered by three effectors (Figure 2A). The differential CPK activation is unlikely due to the differences in the expression levels and timing of effector expression (Figure S2B). In light of the observation that AvrRpm1/AvrB-RPM1 interaction triggers a more rapid cell death than the AvrRpt2-RPS2 interaction (Figure 1A and S1A), we hypothesized that the 72-kDa CPKs were likely involved in regulating PCD. Importantly, effector-mediated kinase activation was not observed in the corresponding *rpm1* and *rps2* mutants (Figure 2B and S2C), reinforcing the requirement for host immune sensors in transducing Ca^2+^ signaling. The weak response mediated by AvrB-TAO1 [40] and AvrRpm1-RPS2 [41] might be below the threshold of detection for CPK activation. The activation of CPKs by bacterial effectors was further confirmed in *Arabidopsis* plants inoculated with *Pst*, *Pst avrRpm1* or *avrRpt2* (Figure 2C). Notably, bacterial flagellin-mediated CPK activation is rather transient and peaks within 5–15 min [33]. In contrast, coincident with sustained cytoplasmic Ca^2+^ elevation, effector-triggered CPK activation lasted for hours (Figure 2A) [25], [26]. In addition, unlike flagellin, AvrRpm1 and AvrRpt2 did not induce strong MAPK activation (Figure 2D and S2D), indicating differential early signaling events in PTI and ETI. Kinase inhibitor K252a and Ca^2+^ channel blockers, LaCl_3_ and RR, substantially abolished the activation of putative CPKs (Figure 2E), further confirming the requirement of Ca^2+^ signaling in the kinase activation. Catalase, a decomposer of H_2_O_2_, or NO scavenger CPTIO and NO synthase inhibitor L-NNA had no effects on the kinase activation (Figure 2E), implying that the CPK activation likely occurs upstream or independently of ROS and NO signaling, which are induced upon *Pst avrRpm1* or *avrRpt2* infection in *Arabidopsis* leaves [24], [26], [42].

### Functional genomic screen of CPKs in ETI signaling

The predicted molecular mass of CPK1 and CPK2 in group I matches the putative 72-kDa CPKs whose activation kinetics was coincident with the onset of effector-triggered PCD, whereas the majority of the remaining CPKs falls into the range of molecular mass of 60-kDa [27]. We reasoned that if any specific CPK functions in ETI signaling, its constitutively active (CPKac) form lacking the autoinhibitory domain [33] would likely activate ETI marker gene *WRKY46* in the absence of effectors. We performed a functional genomic screen by co-expressing individual *CPKac* with *pWRKY46-LUC* in protoplasts. Among the 23 *CPKs* that are well expressed in *Arabidopsis* leaves [33], only specific CPKacs, CPKac3, 4, 5, 6, 10, 11 and 30, induced *pWRKY46-LUC* expression two to four fold (Figure 2F). The expression level and kinase activity of CPKac3 are relatively higher than the other CPKacs [33]. Notably, CPKac4, 5, 6 and 11 belong to a closely related clade in subgroup I [27]. The molecular mass of CPK4, 5, 6, and 11 is around 60 kDa [33], which matches 60-kDa CPKs activated by effectors. Thus, CPK4, 5, 6, and 11 were chosen for the further studies. The kinase-dead mutants of CPKac4, 5 and 11 did not activate *pWRKY46-LUC* expression (Figure 2G). CPKac1 and 2, which are likely involved in PCD regulation, did not significantly induce *pWRKY46-LUC* (Figure 2F).

### WRKY transcription factors act synergistically with CPKs in ETI signaling

**Figure 3:**
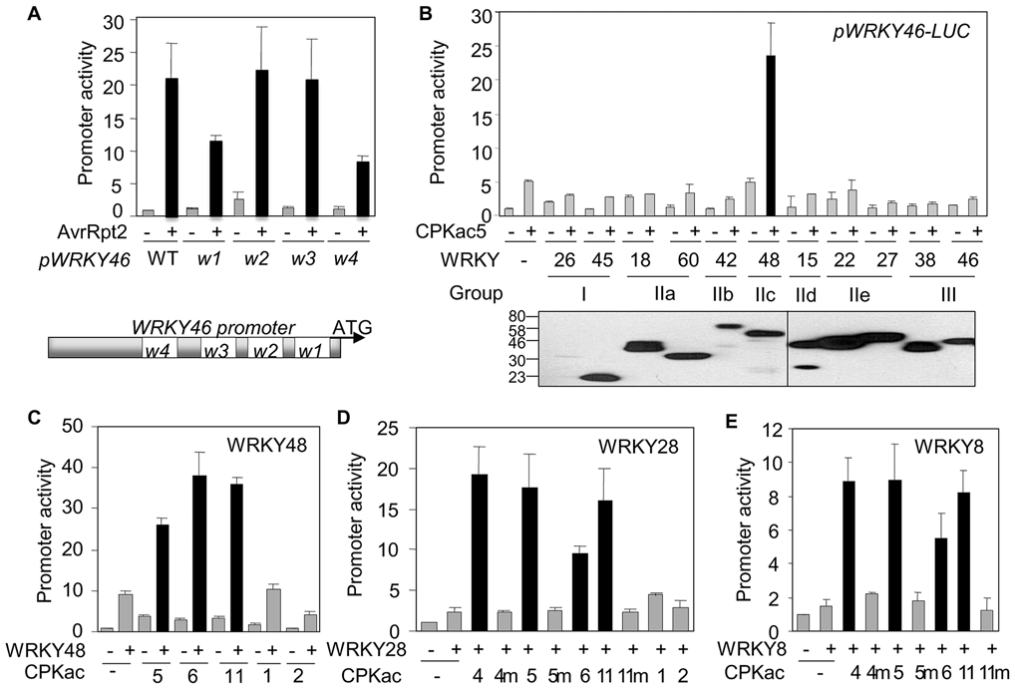
Synergism of CPKs and WRKYs on *WRKY46* promoter activity. (**A**) Requirement of W-boxes for *WRKY46* promoter activity in protoplasts. The WT or mutant *WRKY46* promoter was co-transfected with *avrRpt2* or a vector control. The scheme represents the positions of four W-boxes in the *WRKY46* promoter. (**B**) Functional genomic screen of WRKYs in protoplasts. The representative *WRKY* from different groups were co-transfected with *CPKac5* for the activation of *WRKY46* promoter. The bottom panel shows the expression of individual HA epitope-tagged WRKYs detected by Western blot. (**C**) Synergistic activation of *WRKY46* promoter by WRKY48 and specific CPKacs in protoplasts. (**D**) Synergistic activation of *WRKY46* promoter by WRKY28 and specific CPKacs in protoplasts. “m” indicates the kinase-dead mutants of CPKacs. (**E**) Synergistic activation of *WRKY46* promoter by WRKY8 and specific CPKacs in protoplasts. “m” indicates the kinase-dead mutants of CPKacs. The above experiments were repeated three times with similar results.

Compared to the strong activation by effectors (Figure 1E), CPKacs only moderately activated the *WRKY46* promoter. We hypothesized that additional factors may be involved to act synergistically with CPKs for *WRKY46* promoter activation in ETI signaling. Bioinformatics analysis of the putative promoter region (1.5 Kb upstream of the translational start codon) of *WRKY46* identified four W-box elements that are recognized by WRKY transcription factors (Figure 3A) [43]. Compared to the wild-type reporter, the mutation of W1 or W4 attenuated AvrRpt2-mediated activation of *pWRKY46-LUC* (Figure 3A), suggesting the involvement of WRKYs in ETI signaling.

The 75 *Arabidopsis WRKY* genes were classified into three groups with group II further divided into five subgroups [44]. We carried out a second functional genomic screen to identify WRKY candidates that could function synergistically with specific CPKs in ETI signaling. Representative *WRKYs* induced by *Pst avrRpt2* from each *WRKY* group (Figure S3A) [43] were co-expressed with CPKac5 in protoplasts for the activation of *pWRKY46-LUC* reporter. Remarkably, co-expression of CPKac5 and WRKY48 in subgroup IIc strongly induced the *WRKY46* promoter to the same extent as that activated by effectors (Figure 3B). Consistently, CPKac4, 6 and 11, close family members of CPKac5, but not CPKac1 and 2 that were unable to activate *WRKY46* promoter (Figure 2F), also exhibited synergistic activity with WRKY48 to induce *pWRKY46-LUC* (Figure 3C and S3B). WRKY8 and 28, closely related to WRKY48 in subgroup IIc, also strongly activated *pWRKY46-LUC* when co-expressed with CPKac4, 5, 6 or 11, but not their kinase-dead mutants (Figure 3D and 3E), suggesting potentially overlapping functions of WRKY8, 28 and 48 in ETI signaling. Consistently, the expression of *WRKY8*, *28* and *48* preceded that of *WRKY46* upon *Pst avrRpt2* infection (Fig, S3C). Together, our results indicate that CPK4, 5, 6 and 11 play overlapping or redundant roles in immune gene regulation via specific WRKY transcription factors.

### Direct phosphorylation of WRKYs by CPKs

**Figure 4:**
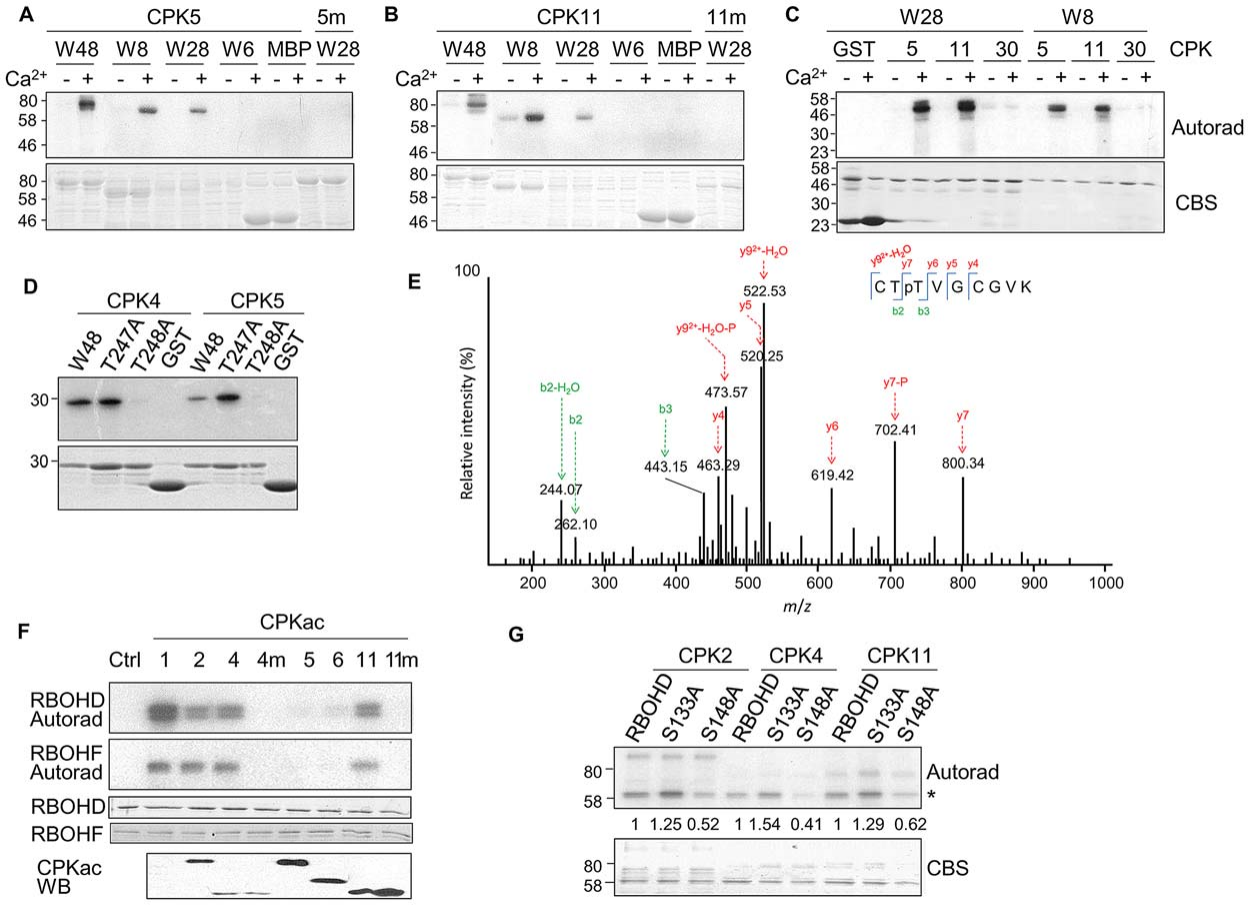
CPKs phosphorylate WRKYs and RBOHs. (**A**) Phosphorylation of WRKYs by CPK5 *in vitro*. MBP-WRKY fusion proteins were used as the substrates for GST-CPK5 in an *in vitro* kinase assay in the presence of 1 mM Ca^2+^. Phosphorylation was analyzed by autoradiography (top panel), and the protein loading was shown by Coomassie blue staining (CBS) (bottom panel). 5 m is a kinase-dead mutant of CPK5. (**B**) Phosphorylation of WRKYs by CPK11 *in vitro*. 11 m is a kinase-dead mutant of CPK11. (**C**) Phosphorylation of WRKY DNA binding domains by different CPKs *in vitro*. (**D**) T248 is required for WRKY48 DNA binding domain phosphorylation by CPKs *in vitro*. (**E**) WRKY48 T248 is phosphorylated by CPKs with MS analysis. Sequencing of a doubly charged peptide ion at m/z 531.22 that matches to CTpTVGCGVK of WRKY48. The confident b2 and b3 ions as well as y7 ion provide strong evidence for phosphorylation of the third Thr residue. (**F**) CPKacs phosphorylated RBOHD and RBOHF with an immunocomplex kinase assay. The FLAG-tagged CPKacs or the kinase-dead mutants (m) were expressed in protoplasts, and immunoprecipitated with an α-FLAG antibody for an *in vitro* kinase assay using GST-RBOHD or GST-RBOHF as a substrate. The proteins of RBOHD and RBOHF were shown, and the expression of individual CPKacs was detected by Western blot (bottom panel). (**G**) S148 is an essential phosphorylation site of RBOHD by CPKs *in vitro*. * indicates phosphorylated RBOHD. The numbers below indicate the relative phosphorylation level compared to WT RBOHD (set as 1) as quantified by Image J. The above experiments were repeated three times with similar results. The MS analysis was repeated twice.

To determine whether CPKs could directly phosphorylate WRKYs for their functional synergism, we purified full-length CPKs as Glutathione-S-Transferase (GST) and WRKYs as Maltose-Binding Protein (MBP) fusion proteins from *E. coli* and carried out *in vitro* kinase assays. Significantly, CPK4, 5 and 11, but not the kinase-dead mutants, were able to phosphorylate WRKY8, 28 and 48 in a Ca^2+^ dependent manner (Figure 4A, 4B and S4A). The conserved DNA-binding WRKY domain of WRKY8, 28 and 48 could be directly phosphorylated by CPK4, 5 and 11, but not by 10 and 30 (Figure 4C, 4D and data not shown). The amino acid sequence surrounding T247 and T248 of WRKY48 [basic-X-T-T-X-X-X-X-hydrophobic (θ)-basic] closely matches an optimal phosphorylation substrate target of CPKs (basic-θ-X-basic-X-X-S/T-X-X-X-θ-basic) [27]. Indeed, both T247 and T248 were phosphorylated by CPKs *in vitro* with mass spectrometry (MS) analysis (Figure 4E and S4B). Interestingly, T248A, but not T247A, abolished the phosphorylation of the WRKY48 DNA binding domain by CPK4 and 5 (Figure 4D), suggesting the functional importance of T248 in WRKY48. T248 in WRKY48 is conserved in WRKY8 and 28 (Figure S3A). Importantly, T199 in WRKY28, corresponding to WRKY48 T248, was also phosphorylated by CPK5 with MS analysis (Figure S4C).

### Phosphorylation of NADPH oxidases by CPKs

ETI signaling is often associated with a rapid production of ROS generated by plasma membrane-resident NADPH oxidases encoded by *RBOH* genes in plants. *Arabidopsis rbohD rbohF* double mutants showed decreased ROS production and PCD in response to *Pst avrRpm1* infection [45]. Potato StCPK4 and 5 phosphorylated StRBOHB and activated ROS production in tobacco leaves [32]. Surprisingly, CPKac5 and 6, the closest orthologs of StCPK4 and 5, only displayed weak phosphorylation activity on the cytoplasmic N-terminus of RBOHD or RBOHF (Figure 4F). However, CPKac1, 2, 4 and 11, but not the kinase-dead mutants, strongly phosphorylated the cytoplasmic N-terminus of RBOHD and RBOHF in an immunocomplex kinase assay (Figure 4F). The weak phosphorylation activity of CPKac5 and 6 on RBOHD and RBOHF was unlikely due to their overall kinase activities (Figure S4D). This finding was further substantiated by the full-length CPK11 phosphorylation of RBOHD and RBOHF in a Ca^2+^-dependent manner with an *in vitro* kinase assay (Figure S4E). StCPKs phosphorylated StRBOHB at residues Ser-82 and Ser-97 [32], corresponding to Ser-133 and Ser-148 in *Arabidopsis* RBOHD. Mutation of Ser-148, but not Ser-133, to alanine reduced the RBOHD phosphorylation by CPK2, 4 and 11 (Figure 4G), indicating Ser-148 as an important phosphorylation site of RBOHD by CPKs. The data suggest that specific *Arabidopsis* CPKs play an important role in ROS production by phosphorylating NADPH oxidases.

### CPK phosphorylation enhances WRKY binding to W-boxes

**Figure 5:**
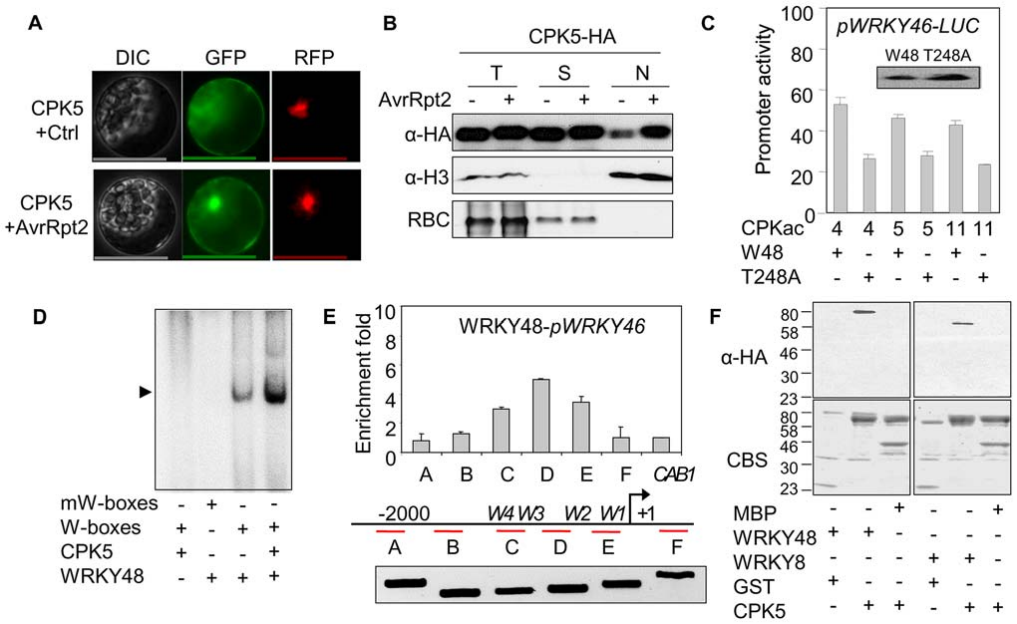
CPKs enhance WRKY binding to the W-boxes. (**A**) Subcellular localization of CPK5 in protoplasts. CPK5-GFP was co-transfected with *avrRpt2* or a vector control, and CPK5-GFP localization was observed with a confocal microscope 12 hpt. The nucleus was indicated with a co-transfected nuclear-localized RFP. Bar = 50 µm. (**B**) Subcellular fractionation of CPK5 in protoplasts. CPK5-HA was co-transfected with *avrRpt2* or a vector control. Total protein extracts (T) were separated into nuclear (N) and soluble (S) fractions. CPK5 expression was detected by Western blot with an α-HA antibody. The purity of the nuclear and soluble fractions was demonstrated with α-Histone H3 antibody and CBS for RuBisCO (RBC). (**C**) T248 was required for WRKY48 synergistic activation with CPKs on *WRKY46* promoter in protoplasts. The protein expression of WRKY48 and its T248A mutant was shown in the insert. (**D**) CPK5 enhanced WRKY48 binding to the W-boxes *in vitro*. The recombinant WRKY48 protein was incubated with ^32^P-labeled W-boxes or mutated W-boxes (mW-boxes) probe in a gel mobility shift assay. CPK phosphorylation of WRKY48 was performed prior to DNA binding assay. (**E**) WRKY48 bound to the endogenous *WRKY46* promoter regions enriched with W-boxes in protoplasts. Fragment A to F were ChIP-PCRed with primers across *WRKY46* promoter and gene body. *W1* to *W4* indicate the positions of W-boxes corresponding to Figure 3A. *CAB1* is a control gene. +1 is the transcriptional start site. Data are shown as mean ± SD. The input control for each primer pair was shown on the bottom. (**F**) *In vitro* pull down of WRKYs and CPK5. MBP was the control for MBP-fused WRKY proteins with a HA tag. GST was the control for GST-fused CPK5 proteins. MBP-WRKY48-HA, MBP-WRKY8-HA or MBP proteins were incubated with GST or GST-CPK5 beads, and the beads were collected and washed for Western blot of immunoprecipitated proteins with an α-HA antibody. The above experiments were repeated three times with similar results.

The GFP fusions of CPK4, 5, 6 and 11 were observed in both cytoplasm and nucleus [33], whereas WRKY8 and 48 were mainly detected in the nucleus [46], [47]. Since CPKs directly phosphorylated WRKYs, we examined the localization of CPK-GFP upon effector elicitation. Interestingly, co-expressing with AvrRpt2 enriched the strong and bright nuclear CPK5-GFP signals (Figure 5A). The enriched nuclear GFP signal was not due to the cleavage of CPK-GFP by AvrRpt2 (Figure S5A). Similarly, expression of AvrRpt2 under the control of a dexamethasone-inducible promoter within 2 hr was able to stimulate both CPK4-GFP (Figure S5B) and CPK5-GFP (Figure S5C) nuclear localization. Subcellular fractionation further confirmed a quantitative increase of CPK5-HA protein in the nucleus in the presence of AvrRpt2 (Figure 5B). The purity of subcellular fractionations was confirmed with α-histone H3 antibody for nuclear proteins and coomassie blue staining of rubisco carboxylase (RBC) for proteins excluded from the nucleus (Figure 5B). The data suggest that AvrRpt2 stimulates CPK5 nuclear translocation, where CPK5 phosphorylates specific WRKYs to regulate target gene transcription. The biological importance of phosphorylation was reinforced by that mutation of T248, a CPK phosphorylation residue in the DNA binding domain of WRKY48, partially compromised its ability to activate *pWRKY46-LUC* in the presence of CPKac4, 5 or 11 (Figure 5C).

WRKYs bind to the W-boxes of target genes to regulate transcription. We show that WRKY48 proteins bound to the DNA oligos consisting of four W-boxes from *WRKY46* promoter in a gel mobility shift assay (Figure 5D and S6A) and quantitative chromatin immunoprecipitation-polymerase chain reaction (ChIP-PCR) assay (Figure 5E). The binding appears specific as WRKY48 proteins did not bind to the mutated W-boxes (Figure 5D), and the binding was largely reduced with the addition of unlabeled specific oligos, but not with nonspecific oligos (Figure S6B). Importantly, phosphorylation of WRKY48 or 28 by CPK5 further enhanced its binding to the W-boxes (Figure 5D and S6C). Apparently, phosphorylation is essential for the enhanced binding activity since the kinase-dead mutant CPK5m did not potentiate WRKY28 binding to the W-boxes (Figure S6C). Consistently, an *in vitro* assay revealed that CPK5 directly pulled down WRKY8 or 48, suggesting a physical interaction between specific WRKYs and CPKs (Figure 5F). Together, the data support the synergistic roles of specific CPKs and WRKYs in *WRKY46* activation in ETI signaling.

### Compromised ETI signaling and pathogen resistance in *cpk* mutants

**Figure 6:**
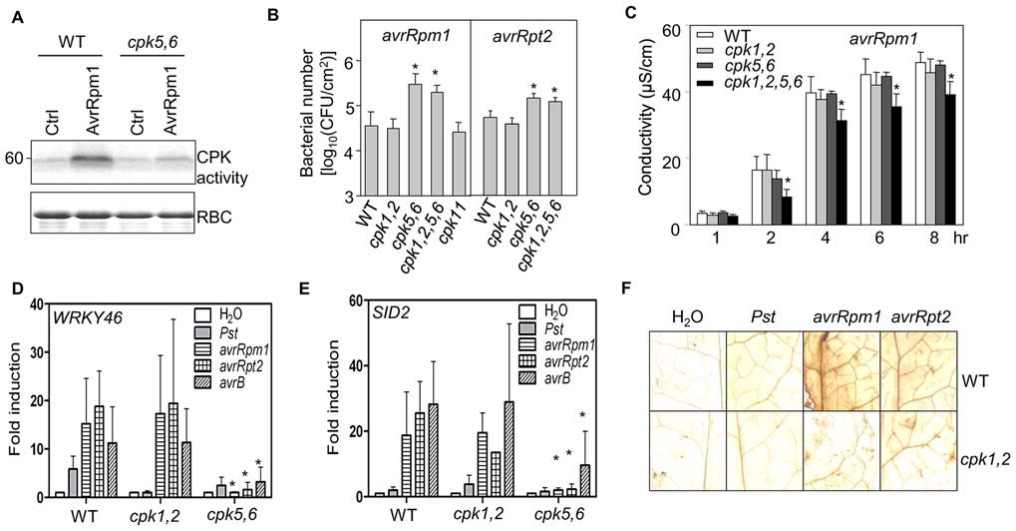
The compromised immune responses in *cpk* mutants. (**A**) Effector-induced WRKY28 phosphorylation was abolished in *cpk5,6* mutant protoplasts. An in-gel kinase assay using fusion protein of MBP-WRKY28 DNA binding domain as a substrate was performed with protoplasts transfected with *AvrRpm1* or a control vector. The equal protein loading was shown by CBS. (**B**) The *cpk5,6* mutant plants were compromised in effector-mediated disease resistance. Plant leaves were hand-inoculated with *Pst avrRpm1* or *avrRpt2* at 5×10^5^ cfu/ml. The bacterial growth was measured 4 dpi. The data are shown as mean ± SE of three repeats, and the asterisk (*) indicates a significant difference with p<0.05 when compared with data from WT plants. (**C**) *Pst avrRpm1*-induced electrolyte leakage in plants. Plant leaves were hand-inoculated with *Pst avrRpm1* at 1×10^8^ cfu/ml, and leaf discs were excised at the indicated time points. The data are shown as the mean ± SE (n = 3) and the asterisk (*) indicates a significant difference between *cpk1,2,5,6* and WT (p<0.05). (**D**) Effector-induced *WRKY46* expression was reduced in *cpk* mutant plants. *WRKY46* expression was detected in plants 6 hr after hand-inoculation with bacteria at 1×10^7^ cfu/ml. The expression of *WRKY46* was normalized to the expression of *UBQ10*. The data are shown as the mean ± SE from three independent biological replicates. * indicates a significant difference with p<0.05 when compared with data from WT plants. (**E**) Effector-induced *SID2* expression was reduced in *cpk* mutant plants. (**F**) H_2_O_2_ production was compromised in the *cpk1,2* mutant plants. The leaves were hand-inoculated with H_2_O, *Pst*, *Pst avrRpm1* and *avrRpt2* at 5×10^7^ cfu/ml, and excised at 24 hpi for DAB staining to detect H_2_O_2_ production. The above experiments were repeated three times with similar results.

To examine the genetic importance of specific *CPKs* in ETI signaling, we characterized *Arabidopsis* loss-of-function *cpk* mutants. In addition to our previously identified *cpk5*, *cpk6* and *cpk11* single mutants and the *cpk5,6* double mutants [33], we isolated *cpk1* (Salk_096452) and *cpk2* (Salk_059237) single mutants from the Salk T-DNA insertion collection (Figure S7A). RT-PCR analysis confirmed that both *cpk1* and *cpk2* were null mutants with undetectable full-length transcripts (Figure S7A). We did not observe overt phenotypes for any single mutants (*cpk1*, *2*, *5*, *6* and *11*) in response to *Pst avrRpm1* or *avrRpt2* infections (data not shown). We further generated the *cpk1,2* double mutants and the *cpk1,2,5,6* quadruple mutants by genetic crosses. These mutants did not display any obvious growth defects under normal growth conditions. Importantly, AvrRpm1-stimulated WRKY28 phosphorylation by endogenous CPKs was reduced in the *cpk5,6* mutants with WRKY28 fusion protein as a substrate in an in-gel kinase assay (Figure 6A).

The *in planta* bacterial multiplication of *Pst avrRpm1* or *avrRpt2* increased about five to ten fold in the *cpk5,6* and *cpk1,2,5,6*, but not *cpk1,2* mutants, compared to that in WT plants (Figure 6B). The disease symptom was also more severe in the *cpk5,6* and *cpk1,2,5,6* mutants than that in WT and *cpk1,2* mutants (Figure S7B). The increased susceptibility of the *cpk5,6* mutants to *Pst avrRpm1* or *avrRpt2* was not due to a general defect in basal defense (Figure S7C). NLR proteins were divided into TIR (Toll-interleukin 1 receptor)-domain-containing and CC (coiled-coil)-domain-containing classes. Interestingly, the *cpk5,6* and *cpk1,2,5,6* mutants were also more susceptible to the infection by *Pst avrRps4*, mediated by TIR-type NLR RPS4 (Figure S7D). Consistently, AvrRps4 activated expression of *WRKY46* promoter (Figure S7E). The data suggested the involvement of CPK5 and 6 in disease resistance mediated by both CC- and TIR-type NLRs. However, the cell death triggered by *Pst avrRpm1* and *avrRpt2* was partially compromised only in the *cpk1,2,5,6*, but not in the *cpk1,2* or *cpk5,6* mutants (Figure S7F). We further quantified PCD using an electrolyte leakage assay. Consistently, compared to WT plants, *cpk1,2,5,6* mutants showed a diminished increase in conductance, due to the release of electrolytes during cell death upon *Pst avrRpm1* infection (Figure 6C). Thus, *CPK5* and *6* play roles in pathogen resistance, whereas *CPK1* and *2* together with *CPK5* and *6* are likely involved in the control of PCD in ETI signaling.

To obtain further genetic evidence of specific CPKs in ETI-mediated transcriptional reprogramming, we examined immune gene expression by pathogen effectors in *cpk* mutants. The *WRKY46* induction by *Pst avrRpm1*, *avrB*, or *avrRpt2* was abolished in the *cpk5,6* mutants, but not *cpk1,2* mutants (Figure 6D), consistent with the role of CPK5 and 6 in phosphorylating specific WRKYs. Similarly, the *WRKY46* transcripts induced by AvrRpm1 or AvrB in protoplasts were reduced in *cpk5,6* mutants (Figure S7G). Infection of plants with *Pst avrRpm1*, *avrB*, or *avrRpt2* also induced strong induction of *SID2* gene, which was diminished in *cpk5,6* mutants (Figure 6E). Consistent with CPK1 and 2 phosphorylating RBOHD and RBOHF *in vitro* (Figure 4F), the ROS production induced by *Pst avrRpm1* or *avrRpt2* was reduced in *cpk1,2* double mutants (Figure 6F). Together, these data provide genetic evidence that Ca^2+^ signaling via specific CPKs plays pivotal roles in the diverse downstream signaling and pathogen resistance mediated by distinct intracellular NLR immune sensors.

### WRKY 8 and WRKY48 as positive regulators in convergent ETI signaling

**Figure 7:**
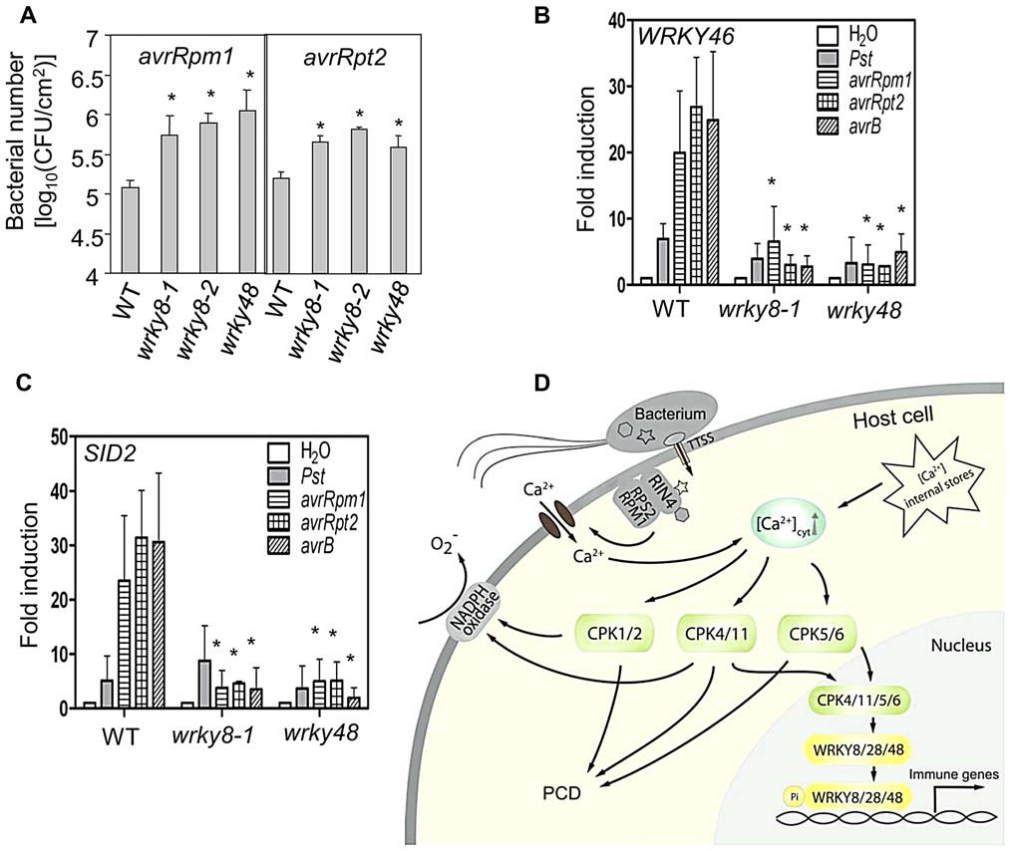
The compromised immune responses in *wrky* mutants. (**A**) The bacterial growth in *wrky8* and *wrky48* mutant plants. Plant leaves were hand-inoculated with *Pst avrRpm1* or *avrRpt2* at 5×10^5^ cfu/ml. The bacterial growth was measured 4 dpi. The data are shown as mean ± SE of three repeats, and the asterisk (*) indicates a significant difference with p<0.05 when compared with data from WT plants. (**B**) Effector-induced *WRKY46* expression was reduced in *wrky* mutant plants. *WRKY46* expression was detected in plants 6 hr after hand-inoculation with bacteria at 1×10^7^ cfu/ml. The expression of *WRKY46* was normalized to the expression of *UBQ10*. The data are shown as the mean ± SE from three independent biological replicates. * indicates a significant difference with p<0.05 when compared with data from WT plants. (**C**) Effector-induced *SID2* expression was reduced in *wrky* mutant plants. (**D**) A model of bifurcate NLR immune signaling via specific and overlapping CPKs. TTSS: type III secretion system. The above experiments were repeated three to four times with similar results.

To reveal the function of WRKYs in ETI signaling, we characterized the loss-of-function *wrky* mutants. The *wrky8-1* (Salk_107668), *wrky8-2* (Salk_050194) and *wrky48* (Salk_066438) mutants are null alleles with undetectable full-length transcripts (Figure S8A) [46], [47], whereas the available T-DNA insertion lines of *wrky28* (Salk_007497 and Salk_092786) mutants did not significantly reduce its transcript level (data not shown). Significantly, the *wrky8-1*, *wrky8-2* and *wrky48* mutants were partially immunocompromised to *Pst avrRpm1*, *avrRpt2* and *avrB* infection. The bacterial population in the *wrky* mutants was about five to ten fold more than that in WT plants 4 days post infection (dpi) (Figure 7A and S8B). The disease symptom was also more pronounced in the *wrky* mutants than that in WT plants (Figure S8C). The *wrky8-1*, *wrky8-2* and *wrky48* mutants did not affect the PCD induced by *Pst avrRpm1* or *avrB* (Figure S8D). Our results suggest that *WRKY8* and *48* play positive roles in plant ETI-mediated disease resistance. These findings are in contrast to the negative regulation of *WRKY8* and *48* in plant basal defense to *Pst* infection (Figure S8E) [46], [47]. Apparently, the same transcription factors may serve distinct functions in plant PTI and ETI signaling or in response to different pathogens.

We further examined immune gene expression by pathogen effectors in *wrky* mutants. The *WRKY46* and *SID2* induction by *Pst avrRpm1*, *avrB*, or *avrRpt2* was diminished in the *wrky8-1* and *wrky48* plants (Figure 7B and 7C). Similarly, the effector-mediated activation of *WRKY46* transcripts was reduced in the *wrky8-1* and *wrky48* protoplasts (Figure S8F). The physiological and genetic analyses with *cpk* and *wrky* mutants thus substantiate the specific and overlapping functions of CPKs in phosphorylating distinct substrates for the bifurcate control of immune gene activation, PCD and ROS production (Figure 7D).

## Discussion

Plants have evolved sophisticated innate immune systems to effectively defend pathogen attacks without specialized immune cells and the adaptive immune system. Polymorphic plant NLR R proteins are intracellular immune sensors that recognize pathogen-encoded effectors to initiate complex immune responses, including a sustained increase in cytosolic Ca^2+^ concentration, transcriptional reprogramming, production of ROS, and PCD. Recent studies have advanced our understanding of NLR protein functions in terms of effector recognition, subcellular localization and structural determination, but the molecular mechanisms leading to the convergent immune responses upon NLR activation remain enigmatic [8], [9], [11], [48]. In this study, we uncovered the molecular consequences of sustained Ca^2+^ elevation, which leads to bifurcate signaling events controlled by specific and overlapping CPKs through phosphorylation of distinct substrates upon NLR protein activation. Two major groups of CPKs were dynamically activated by bacterial effectors AvrRpm1, AvrB and AvrRpt2. Functional genomic and biochemical analyses revealed that CPK4, 5, 6 and 11 were involved in immune gene activation, whereas CPK1 and 2, and likely 4 and 11 played key roles in the control of ROS generation, and CPK1, 2, 4, 5, 6 and 11 together contributed to PCD. CPK4, 5, 6 and 11 phosphorylated WRKY8, 28 and 48, leading to enhanced WRKY protein binding to the W-boxes of specific target gene promoters for transcriptional regulation, whereas CPK1, 2, 4 and 11 *in vitro* phosphorylated RBOHD and RBOHF for ROS production. Genetic and physiological characterization of multiple knockout mutants substantiated the biochemical data as *cpk5,6*, *wrky8* and *wrky48* mutants were compromised in immune gene activation and disease resistance, *cpk1,2* mutants were impaired in effector-induced oxidative burst and *cpk1,2,5,6* mutants were defective in PCD. Taken together, our studies decode the specific functions of individual CPKs in the control of differential ETI responses (Figure 7D). Our findings offer a potential molecular link for the uncoupled PCD and restriction of pathogen growth upon NLR activation [11], [19], [20], [49].

The rapid increase of cytosolic Ca^2+^ concentration has been observed in plants response to MAMPs or pathogen effectors [50]. Apparently, each signal elicits a specific calcium signature with unique kinetics, magnitude, duration and cellular compartment distribution. MAMPs, such as flagellin and PGN, activate Ca^2+^ increase for 5–15 min [34], coincident with transient CPK activation [33]. However, *Pst avrRpm1* or *avrB* elicited a Ca^2+^ transient increase with a maximum about 10 min followed by a sustained increase peaked around 2 hr after infection [25]. Treatment of La^3+^, Gd^3+^ and RR significantly suppressed AvrRpm1- and AvrRpt2-mediated gene activation and cell death (Figure 1F, 1G and S2A), indicating that both extracellular and intracellular Ca^2+^ release contributes to ETI signaling. It has been suggested that cyclic nucleotide-gated channels (CNGCs) function in conducting Ca^2+^ to mediate PCD [24], [26]. Interestingly, *Arabidopsis dnd (defense no death)* and *hlm1 (hr-like lesion mimic)* mutants, carrying mutations in *CNGC2* and *CNGC4* genes, exhibited aberrant PCD depending on genetic backgrounds and growth conditions [51], [52], [53]. The constitutive *PR1* activation and enhanced pathogen resistance in the *dnd* and *hml1* mutants may be a consequence of low intrinsic Ca^2+^ levels due to *CNGC* mutations. It will be interesting to determine whether specific CNGCs are responsible for CPK-WRKY activation and the immune gene induction. Future studies may elucidate the precise functions of various CNGCs and other Ca^2+^ channels in mediating distinct Ca^2+^ signatures of extracellular and internal origins upon NLR activation.

WRKYs are a group of plant specific transcription factors involved in transcriptional reprogramming during various biological processes, in particular plant defense responses [44]. A large number of the *Arabidopsis WRKY* genes are transcriptionally activated upon pathogen infection [43]. Genetic analyses have indicated many WRKYs function as negative regulators in plant defense. For example, *WRKY11* or *17* loss-of-function rendered plant more resistant to *Pst* infection [54]. Similarly, *wrky8* or *48* mutants were more resistant, while overexpressors were more susceptible to *Pst* infection [46], [47]. Despite unclear molecular mechanism of WRKY8 and 48 in plant basal defense, it is likely that WRKY8 and 48 act as repressors of plant PTI signaling. Surprisingly, our results suggest that WRKY8 and 48 play positive roles in ETI signaling since the *wrky8* or *48* mutants were compromised in effector-mediated disease resistance and defense gene activation (Figure 7A, 7B and 7C). Consistently, *WRKY8*, *28* and *48* were quickly and strongly activated upon *Pst avrRpt2* infection independently or upstream of SA signaling [43]. The distinct functions of WRKYs in PTI and ETI signaling could be regulated at transcriptional, translational and post-translational levels in response to different stimuli. Alternatively, differential phosphorylation events mediated by distinct kinases could modulate the different immune responses in PTI and ETI signaling.

It has been suggested that PTI and ETI share downstream signaling machineries and hormonal networks [55]. Genome-wide gene expression profiling suggests that CPK4, 5, 6 and 11 mediate convergent signaling triggered by multiple MAMPs [33]. Our current study also revealed the involvement of these CPKs in ETI signaling. However, a transient Ca^2+^ increase and CPK activation were observed upon MAMP treatment, whereas effectors induced sustained CPK activation (Figure 2A) [33]. Thus, the timing, amplitude and duration of differential CPK activities appear to dictate their substrate specificity and differential transcriptional reprogramming in ETI and PTI signaling. MAPK activation is a convergent MAMP signaling event [2]. MAPKs play pivotal roles and also act in parallel or synergistically with CPKs in the control of early MAMP responsive genes [33]. However, the role of MAPK cascade in ETI signaling remains unclear. We observed a strong activation of CPKs but little MAPK activation by bacterial effectors in a gene-for-gene dependent and cell-autonomous manner (Figure 2A, 2B, 2C and 2D), suggesting a predominant role of CPKs in ETI signaling mediated by RPM1 and RPS2 in *Arabidopsis*. It is possible that elevated CPK signaling may compromise MAPK activation in ETI signaling [56]. Nevertheless, the current data imply that activation of distinct PRRs, namely cell-surface receptor kinases recognizing MAMPs and intracellular NLR proteins recognizing pathogen-encoded effectors, initiates differential early signaling events, which trigger both overlapping and specific immune responses to maximize plant defense against pathogen attacks.

## Materials and Methods

### Plant growth conditions, chemical treatments and bacterial inoculation

*Arabidopsis* wild-type (Col-0), *cpk* and *wrky* mutant plants were grown in pots containing soil (Metro Mix 360 ) in a growth room at 23°C, 60% relative humidity and 75 µE m^−2^ s^−1^ light with a 12 hr photoperiod for approximately 4 weeks before protoplast isolation or bacterial inoculation. T-DNA insertion mutants *cpk1* (Salk_096452), *cpk2* (Salk_059237), *wrky8-1* (Salk_107668), *wrky8-2* (Salk_050194) and *wrky48* (Salk_066438) were obtained from Arabidopsis Biological Resource Center (ABRC), and confirmed by PCR and RT-PCR analyses. The higher order *cpk* mutants were generated by genetic crosses.

Different *Pst* DC3000 strains were grown overnight at 28°C in the KB medium containing rifamycin (50 µg ml^−1^) or in combination with kanamycin (50 µg ml^−1^). Bacteria were pelleted by centrifugation, washed, and diluted to the desired density. The leaves were hand-inoculated with bacteria using a needleless syringe, collected at the indicated time for bacterial counting or for RNA isolation. To measure bacterial growth, two leaf discs were ground in 100 µl H_2_O and serial dilutions were plated on KB medium with appropriate antibiotics. Bacterial colony forming units (cfu) were counted 0, 2 or 4 days post incubation (dpi) at 28°C. Each data point is shown as triplicates.

At least three independent repeats were performed for all experiments. The representative data with similar results were shown. The statistic analysis was performed using the general linear model of SAS (SAS Institute, Inc., Cary, NC) with mean separations by least significant difference (LSD).

### Protoplast transient assay and identification of WRKY46 as a marker gene in ETI signaling

Protoplast isolation and transient expression assay were conducted as described [37]. In general, protoplasts were collected 6 hpt for promoter activity, protein expression and kinase assays. For reporter assay, *UBQ10-GUS* was co-transfected as an internal transfection control, and the promoter activity was presented as LUC/GUS ratio. Protoplasts transfected with empty vector were used as effector controls.

To identify early immune genes in ETI signaling, 5 ml protoplasts at a density of 2×10^5^/ml were transfected with 500 ul AvrRpm1, AvrRpt2 or a control vector (2 ug/ul). The protoplasts were collected 3 hrs after transfection for RNA isolation, cDNA and cRNA synthesis. The cRNA was fragmented for Affymetrix GeneChip (ATH1) hybridization, washing, staining and scanning at Partners HealthCare Center for Personalized Genetic Medicine (Boston, MA). Data analyses with Affymetrix GeneChip Operating Software (GCOS) and GeneSpring identified *WRKY46* as one of the highest induced genes by *avrRpm1* and *avrRpt2* in two independent biological repeats.

### Plasmid construction, recombinant protein isolation and kinase assays

*Arabidopsis CPK* and *WRKY* genes were amplified by PCR from Col-0 cDNA, and introduced into a plant expression vector with an HA or FLAG epitope-tag at the C terminus. Point mutations of *pWRKY46-LUC*, *WRKY8*, *WRKY28* and *WRKY48* were generated by a site-directed mutagenesis kit (Stratagene). The primer sequences for cloning and point mutations are listed in Table S1.

Different CPKs and WRKY constructs were sub-cloned into a modified GST pGEX4T-1 (Pharmacia) or MBP fusion protein expression vector pMAL-C2 (New England BioLabs) with BamHI and StuI digestion and transformed into *E. coli* strain BL21 (DE3). Expression of GST and MBP fusion proteins and affinity purification were performed with standard protocol, and *in vitro* kinase assay was carried out as described [57]. Immunocomplex kinase assay was conducted as described [37].

### MS analysis

The *in vitro* phosphorylation for MS analysis was performed in a 10 µL reaction containing 20 mM Tris·HCl, pH 7.5, 10 mM MgCl_2_,100 mM NaCl, 3 mM CaCl_2_, 1 mM DTT and 0.1 mM ATP. The fusion proteins of 1 µg CPK4 and 1 µg CPK5 were used to phosphorylate 10 µg of GST fusion proteins of WRKY48 DNA binding domain, and 1 µg CPK5 was used to phosphorylate 10 µg of MBP fusion proteins of WRKY28 DNA binding domain. The reaction was performed for 3 hr at room temperature with gentle shaking, and stopped by adding 4× SDS loading buffer. Six individual reactions were combined and separated by 10% SDS-PAGE gel. The gel was stained with Thermo GelCode Blue Safe Protein Stain and distained with dH_2_O. The corresponding bands were cut for MS analysis, which was performed according to Avila et al. [58]. Briefly, gel bands were in-gel digested with trypsin overnight, and phosphopeptides were enriched for liquid chromatography-MS/MS analysis with a LTQ Orbitrap XL mass spectrometer (Thermo Scientific). The MS/MS spectra were analyzed with Mascot (Matrix Science; version 2.2.2), and the identified phosphorylated peptides were manually inspected to ensure confidence in phosphorylation site assignment.

### CPK in-gel kinase assay

200 ul protoplasts were transfected with 20 ul effector DNA (2 ug/ul), and incubated at RT for 2–6 hr. Protoplasts were lysed in 25 µl of extraction buffer (50 mM Hepes-KOH [pH 7.6], 2 mM EDTA, 10 mM β-glycerophosphate, 20% glycerol, 1 mM Na_3_VO_4_, 1 mM NaF and 1% triton X-100). Protoplast exacts with equal amount of protein were fractioned in a 10% SDS-polyacrylamide gel with 0.25 mg/ml histone type III-S (Sigma). The gel was washed three times for 1 hr with washing buffer (25 mM Tris-HCl [pH 7.5], 0.5 mM DTT, 5 mM NaF, 0.1 mM Na_3_VO_4_, 0.5 mg/ml BSA and 0.1% triton X-100), and then incubated for 18 hr with three changes of renaturation buffer (25 mM Tris-HCl [pH 7.5], 0.5 mM DTT, 5 mM NaF, 0.1 mM Na_3_VO_4_). After equilibration of the gel for 30 min in the reaction buffer (25 mM Tris-HCl [pH 7.5], 0.2 mM CaCl_2_, 12 mM MgCl_2_, 1 mM DTT and 0.1 mM Na_3_VO_4_), the kinase reaction was performed for 1 hr in the reaction buffer with 50 µCi [γ-^32^P] ATP. The reaction was stopped and washed 6 times by 5% TCA and 1% sodium pyrophosphate for 6 hr. The gel was dried and visualized by autoradiography.

### Plant cell death assays

For hypersensitive response (HR) assays, the leaves of 4-week-old plants were hand-inoculated with different bacteria at 1×10^8^ cfu/ml, and the cell death for each genotype was calculated as the percentage of leaves showing typical HR response to total leaves inoculated.

For trypan blue staining, leaves were collected 8 hpi for *Pst avrRpm1* and 16 hpi for *Pst avrRpt2*, and stained with trypan blue in lactophenol (Lactic acid∶ glycerol∶ liquid phenol∶distilled water = 1∶1∶1∶1) solution. The stained leaves were destained with 95% ethanol/lactophenol solution, and washed with 50% ethanol. For electrolyte leakage assays, eight leaf discs (0.5 cm diameter) were excised from the WT or *cpk* mutants infiltrated with bacteria and pre-floated in 10 ml of ddH_2_O for 10–15 min to eliminate wounding effect. The ddH_2_O was then exchanged and electrolyte leakage was measured using a conductivity meter (VWR; Traceable Conductivity Meter) with three replicates per time point per sample (n = 8). The YO-PRO-1 iodide was purchased from Molecular Probes/Invitrogen.

### Electrophoretic mobility shift assay

Electrophoretic mobility shift assay (EMSA) was conducted as described [47] with modifications. Briefly, a pair of complementary single-stranded synthetic oligonucleotides (1.25 µM each) was end-labeled at 37°C with [γ-^32^P] ATP for 1 hr using T4 DNA polynucleotide kinase. The labeled oligonucleotides were mixed and annealed in TE buffer (pH 7.5) with 0.1 M NaCl at 65°C for 15 min, followed by gradual cooling to room temperature. After annealing, the double-stranded oligonucleotide probes were purified with QIAquick Nucleotide Removal kit (Qiagen). Binding reaction contains 1 µl of poly-dIdC (Roche) at 1 µg/µl, 2 µl of 5× Binding buffer (4% glycerol, 1 mM MgCl_2_, 0.5 mM EDTA, 0.5 mM DTT and 10 mM Tris-HCl, pH 7.5), 1 µl of labeled probe (approximately 20,000 cpm), 1 µl cold competitor (if needed), 0.1 µl 100× BSA (10 mg/ml) and 2.5 µg recombinant proteins. DNA-protein complexes were allowed to form at room temperature for 30 min and resolved on a 5% native polyacrylamide gel in 0.5× TBE. The gel was dried and exposed on X-ray. For the effect of CPK phosphorylation on WRKY binding activity, the MBP-WRKY proteins were subjected to the phosphorylation assay by CPKs for 1 hr prior to EMSA.

### Detection of ROS production

Histological H_2_O_2_ production in WT and *cpk* mutants upon infection with different *Pst* strains was examined according to the DAB staining method [59] with modifications. Briefly, WT and *cpk* mutant leaves were hand-inoculated with different *Pst* strains at 5×10^7^ cfu/ml for 24 hr. The leaves were excised and subsequently immersed in 1 mg/ml DAB (3,3′-diaminobenzidine, Sigma) (pH 3.8) solution with low vacuum pressure for 30 min, followed by an overnight incubation at room temperature in the dark. The stained leaves were fixed and cleared in alcoholic lacto-phenol (95% ethanol ∶ lactic acid ∶ phenol = 2 ∶ 1 ∶ 1) at 65°C, rinsed once with 50% ethanol, and twice with H_2_O. The destained leaves were stored in 50% glycerol or subjected to microscope observation.

### Subcellular localization and nuclear fractionation

C-terminal GFP fusion of CPK5 was co-transfected with a vector control or *avrRpt2*. Protein localization was observed 12 hpt with a confocal microscopy. The nucleus was indicated with a co-transfected nuclear-localized RFP.

The transfected protoplasts (2 ml at a concentration of 4×10^5^/ml) were lysed with 1 ml extraction buffer (20 mM Tris-HCl, pH 7.0, 25% glycerol, 250 mM sucrose, 20 mM KCl, 1 mM EDTA, 5 mM spermidine, 30 mM β-mercaptoethanol, 1× cocktail protease inhibitors and 1% Triton X-100), and incubated on ice for 10–15 min. The cytoplasmic and nuclear fractions were separated by centrifugation at 1000 g for 10 min at 4°C. The cytoplasmic fraction was aliquoted and frozen at −80°C. The nuclear fraction was washed three times with the nuclei resuspension buffer (20 mM Tris-HCl, pH 7.0, 25% glycerol, 2.5 mM MgCl_2_, 1 mM EDTA, 5 mM spermidine, 30 mM β-mercaptoethanol, 1× cocktail inhibitors, and 0.5% Triton X-100), and resuspended in 20 µl resuspension buffer.

### In vitro pull down assay

HA tagged MBP-WRKY48, MBP-WRKY8 and MBP proteins were pre-incubated with 5 µl prewashed glutathionine agrose beads (Sigma) in 150 µl incubation buffer (10 mM Hepes, pH 7.5, 100 mM NaCl, 1 mM EDTA, 10% glycerol, and 0.5% Triton X-100) at 4°C for 1 hr with gentle shaking. After spinning down at 13,000 rpm for 5 min, the supernatant was transferred and incubated with prewashed GST, GST-CPK5 beads at 4°C for another 1 hr in the presence of 1 mM CaCl_2_. The beads were collected and washed four times with washing buffer (10 mM Hepes, pH 7.5, 100 mM NaCl, 1 mM EDTA, 10% glycerol, and 0.1% Triton X-100) and once with 50 mM Tris·HCl, pH 7.5. The immunoprecipitated proteins were analyzed by Western blot with an α-HA antibody.

### Real-time RT-PCR

Total RNA was isolated from leaves or protoplasts after treatment with TRIzol Reagent (Invitrogen). Complementary DNA was synthesized from 1 µg of total RNA with 0.1 µg oligo (dT) primer and reverse transcriptase (New England BioLabs). Real-time RT-PCR analysis was carried out using iTaq SYBR green Supermix (Bio-Rad) supplemented with ROX in an ABI GeneAmp PCR System 9700. The expression of immune genes was normalized to the expression of *UBQ10*. The primer sequences of different effectors and RT-PCR are listed in Table S1.

### Protoplast ChIP assays

5 ml of protoplasts were transfected with *WRKY48-HA* or *WRKY8-HA* and incubated for 4 hrs. Cells were crosslinked with 1% formaldehyde for 20 min and quenched by glycine for 5 min. Nuclei were extracted freshly as described [60] and the rest of ChIP was performed as described (http://sites.bio.indiana.edu/~pikaardlab/Protocols%20page.html) with some modifications. Bioruptor (Diagenode) was used for sonication and DNA was eluted with 1% SDS and 0.1 M NaHCO_3_ at 65°C for overnight. Anti-HA antibody (Roche) was used. The quantitative PCR primers have similar efficiency. The relative enrichment fold changes were calculated by normalizing % input of each primer pair against the control gene primer (*CAB1*).

## Supporting Information

Figure S1 **Effector induced cell death and gene activation in protoplasts and plants.** (**A**) Hypersensitive response (HR)-induced by *Pst avrRpm1* and *avrRpt2* in plants. *Arabidopsis* leaves were inoculated with bacteria at 1×10^8^ cfu/ml. HR was indicated with the percentage of wilting leaves of total inoculated leaves (n>20) at the different time points after inoculation. *Pst* inoculation was used as a control. (**B**) Effector-induced cell death and nuclear fragmentation detected by YO-PRO-1 iodine staining at 16 hpt in protoplasts. (**C**) AvrRpm1, AvrB and AvrRpt2 activated endogenous *WRKY46* expression in protoplasts. The transfected protoplasts were collected 3 hpt for RT-PCR analysis. The expression of *Actin* was used as a control. (**D**) Induction of *WRKY46* expression in dexamethasone (DEX)-inducible *avrRpt2* transgenic plants and protoplasts. The *WRKY46* expression was detected 3 hr after DEX treatment.
(TIF)

Figure S2 **Ca^2+^ signaling in effector-triggered immunity.** (**A**) *Pst avrRpm1* and *avrRpt2*-induced cell death was suppressed by LaCl_3_ or RR treatment in plants. *Arabidopsis* leaves were inoculated with bacteria at 1×10^8^ cfu/ml in the presence of 2 mM LaCl_3_ or 20 µM RR. The cell death was shown by Trypan blue staining and % indicates the percentage of wilting leaves of total inoculated leaves (n>20). (**B**) Expression of effectors in *Arabidopsis* protoplasts. HA epitope tagged AvrRpt2, AvrRpm1 or AvrB was transfected in protoplasts and cells were collected at the indicated time for Western blot. To avoid cell death, AvrRpt2 was expressed in *rps2*, and AvrRpm1 and AvrB were expressed in *rpm1* mutant protoplasts. (**C**) AvrRpt2-mediated CPK activation depended on RPS2 in protoplasts. The in-gel kinase assay using histone type III-S as substrate was performed 3 hpt. (**D**) Differential activation of MAPKs by flagellin and effectors in protoplasts. Ctrl, *avrRpm1*, or *avrRpt2*-transfected cells were incubated for 1 or 2 hr before the treatment with 1 µM flg22 for 10 min and subjected for an in-gel kinase assay using MBP as substrate.
(TIF)

Figure S3 **CPK and WRKY on** ***WRKY46*** **promoter activity.** (**A**) Alignment of DNA binding domains of WRKYs used in this study. The green box indicates the conserved Threonine (T) residue in WRKY48, 8 and 28. (**B**) Synergism of CPK4 and WRKYs on *WRKY46* promoter activity in protoplasts. The representative WRKYs from different groups were co-transfected with CPKac4 for the activation of *WRKY46* promoter. (**C**) Induction of *WRKY8*, *48*, *28* and *46* by *Pst* and *Pst avrRpt2* at 2 hpi in plants. Plant leaves were hand-inoculated with control or bacteria at 2×10^7^ cfu/ml. The samples were collected 2 hpi for real-time RT-PCR analysis. The expression of *WRKY8*, *48*, *28* and *46* was normalized to the expression of *UBQ10*. The data are shown as the mean ± SE from three repeats.
(TIF)

Figure S4 **Phosphorylation of WRKY and RBOH by CPKs.** (**A**) Phosphorylation of WRKYs by CPK4 *in vitro*. The recombinant MBP fusion proteins of WRKY8, 28 and 48 were used as the substrates for GST-CPK4 in an *in vitro* kinase assay in the presence of 1 mM Ca^2+^. (**B**) MS analysis identified WRKY48 T247 as a phosphorylation site by CPKs. Sequencing of a doubly charged peptide ion at m/z 531.21 that matches to CpTTVGCGVK of WRKY48. The confident b2 and b3 ions as well as y7 ion provide strong evidence for phosphorylation of the second Thr residue. (**C**) MS analysis identified WRKY28 T199 as a phosphorylation site by CPK5. Sequencing of a triply charged peptide ion at m/z 406.84 that matches to CTpTQKCNVK of W28. The confident b3 ion as well as y7^2+^ ion provide strong evidence for phosphorylation of the third Thr residue. (**D**) Phosphorylation activity of CPKacs and CPKs on histone type III-S *in vitro*. FLAG-tagged CPKacs or WT CPKs were expressed in protoplasts and immunoprecipitated with α-FLAG antibody. The kinase activity was determined by *in vitro* assay using histone as a substrate. (**E**) Phosphorylation of RBOHD and RBOHF by CPK11 *in vitro*. The *in vitro* kinase assay was conducted in the presence of 1 mM Ca^2+^. BAK1, the kinase domain of receptor kinase BAK1, was used to show phosphorylation specificity.
(TIF)

Figure S5 **Effector AvrRpt2 stimulates CPK nuclear localization.** (**A**) Expression of CPK4-GFP and CPK5-GFP in the presence of AvrRpt2-HA in protoplasts. Protoplasts were co-transfected with CPK4-GFP or CPK5-GFP and a vector control or AvrRpt2-HA, and expressed for 12 hrs. CPK expression was detected by Western blot with an α-GFP antibody, and AvrRpt2 expression was detected by an α-HA antibody. (**B**) AvrRpt2 stimulates CPK4-GFP nuclear localization in protoplasts. Protoplasts were co-transfected with CPK4-GFP and a vector control (Ctrl) or pTA7001-DEX-AvrRpt2. After expression for 10 hrs, the cells were treated with 10 µM of DEX for 2 or 3 hrs prior to observation of GFP localization. Bar = 50 µm. (**C**) AvrRpt2 stimulates CPK5-GFP nuclear localization in protoplasts.
(TIF)

Figure S6 **Specificity of WRKYs binding to the W-boxes.** (**A**) Sequences of WT W-boxes probe and mutant W-boxes probe (mW-boxes). The W-box sequences corresponding to the *WRKY46* promoter are underlined, and nucleotides in WT probe in blue were mutated in the mutant probe and colored in red. (**B**) Specificity of WRKY48 binding to the W-boxes *in vitro*. The recombinant WRKY48 protein was incubated with ^32^P-labeled W-boxes in a gel mobility shift assay. Specific competitor (S. C.) was non-labeled W-boxes oligonucleotide. Non-specific competitor (N.C.) was a random oligonucleotide. (**C**) Kinase activity is required for CPK-enhanced WRKY28 binding to the W-boxes *in vitro*. CPK phosphorylation of WRKY28 was conducted prior to DNA binding assay.
(TIF)

Figure S7 **Analysis of** ***cpk*** **mutants.** (**A**) T-DNA insertion sites and RT-PCR analysis in *cpk1* and *cpk2* mutants. (**B**) The disease phenotype of WT and *cpk* mutant plant by *Pst avrRpm1* or *avrRpt2* infection. Plant leaves were hand-inoculated with bacteria at 5×10^5^ cfu/ml. The picture was taken at 5 dpi. (**C**) The *cpk5,6* mutant plants were compromised in *avrRpm1-* and *avrRpt2*-mediated disease resistance. Plant leaves were hand-inoculated with *Pst, Pst avrRpm1* or *Pst avrRpt2* at 5×10^5^ cfu/ml. The bacterial growth was measured 2 dpi. The data are shown as mean ± SE of three repeats, and the asterisk (*) indicates a significant difference with p<0.05 when compared with data from WT plants. (**D**) The *cpk5,6* mutant plants were compromised in *avrRps4*-mediated disease resistance. Plant leaves were hand-inoculated with *Pst avrRps4* at 5×10^5^ cfu/ml. The bacterial growth was measured 3 dpi. The data are shown as mean ± SE of three repeats, and the asterisk (*) indicates a significant difference with p<0.05 when compared with data from WT plants. (**E**) AvrRps4 activated *WRKY46* promoter in protoplasts. The p*WRKY46-LUC* was co-transfected with AvrRpm1, AvrRps4 or a vector control in protoplasts and samples were collected at 6 hpt. The *UBQ-GUS* was included as an internal transfection control. The relative luciferase activity was normalized with GUS activity. (**F**) The *cpk1,2,5,6* mutant plants diminished effector-mediated cell death. Plant leaves were hand-inoculated with *Pst avrRpm1* or *avrRpt2* at 1×10^8^ cfu/ml. The cell death ratio was recorded for *avrRpm1* at 8 hpi and *avrRpt2* at 16 hpi. The leaves were further stained with trypan blue to detect cell death. (**G**) Effector-induced *WRKY46* expression was reduced in *cpk* mutant protoplasts. *WRKY46* expression was detected in protoplasts 3 hpt by real-time RT-PCR analysis. The expression of *WRKY46* was normalized to the expression of *UBQ10*. The data are shown as the mean ± SE from three independent biological replicates.
(TIF)

Figure S8 **Analysis of** ***wrky*** **mutants.** (**A**) T-DNA insertion sites and RT-PCR analysis in *wrky8* and *wrky48* mutants. (**B**) The bacterial growth of *Pst avrB* in *wrky* mutant plants. Plant leaves were hand-inoculated with *Pst avrB* at 5×10^5^ cfu/ml. The bacterial growth was measured at 3 dpi. The data are shown as mean ± SE of three repeats, and the asterisk (*) indicates a significant difference with p<0.05 when compared with data from WT plants. (**C**) The disease phenotype of WT and *wrky* mutant plants by *Pst avrRpm1* or *avrRpt2* infection. Plant leaves were hand-inoculated with different bacteria at 5×10^5^ cfu/ml and the pictures were taken at 6 dpi. (**D**) The cell death of *wrky* mutant plants. Plant leaves were hand-inoculated with *Pst avrRpm1* or *avrB* at 1×10^8^ cfu/ml. The cell death ratio was recorded at 10 hpi, and indicated with the percentage (%) of wilting leaves of total inoculated leaves. (**E**) The *wrky* mutant plants are resistant to *Pst* infection. Plant leaves were hand-inoculated with *Pst* at 5×10^5^ cfu/ml. The bacterial growth was measured at 3 dpi. The data are shown as mean ± SE of three repeats, and the asterisk (*) indicates a significant difference with p<0.05 when compared with data from WT plants. (**F**) Effector-induced *WRKY46* expression was reduced in *wrky* mutant protoplasts. *WRKY46* expression was detected in protoplasts 3 hpt by real-time RT-PCR analysis. The expression of *WRKY46* was normalized to the expression of *UBQ10*. The data are shown as the mean ± SE from three independent biological replicates.
(TIF)

Table S1 **Primers used in this study.**
(DOC)
