# Supplementary figures and images for: Bifurcation of Arabidopsis NLR Immune Signaling via Ca2+-Dependent Protein Kinases
Source: PLoS Pathog. 2013 Jan 31;9(1):e1003127. doi: 10.1371/journal.ppat.1003127 (PMC3561149; doi:10.1371/journal.ppat.1003127)

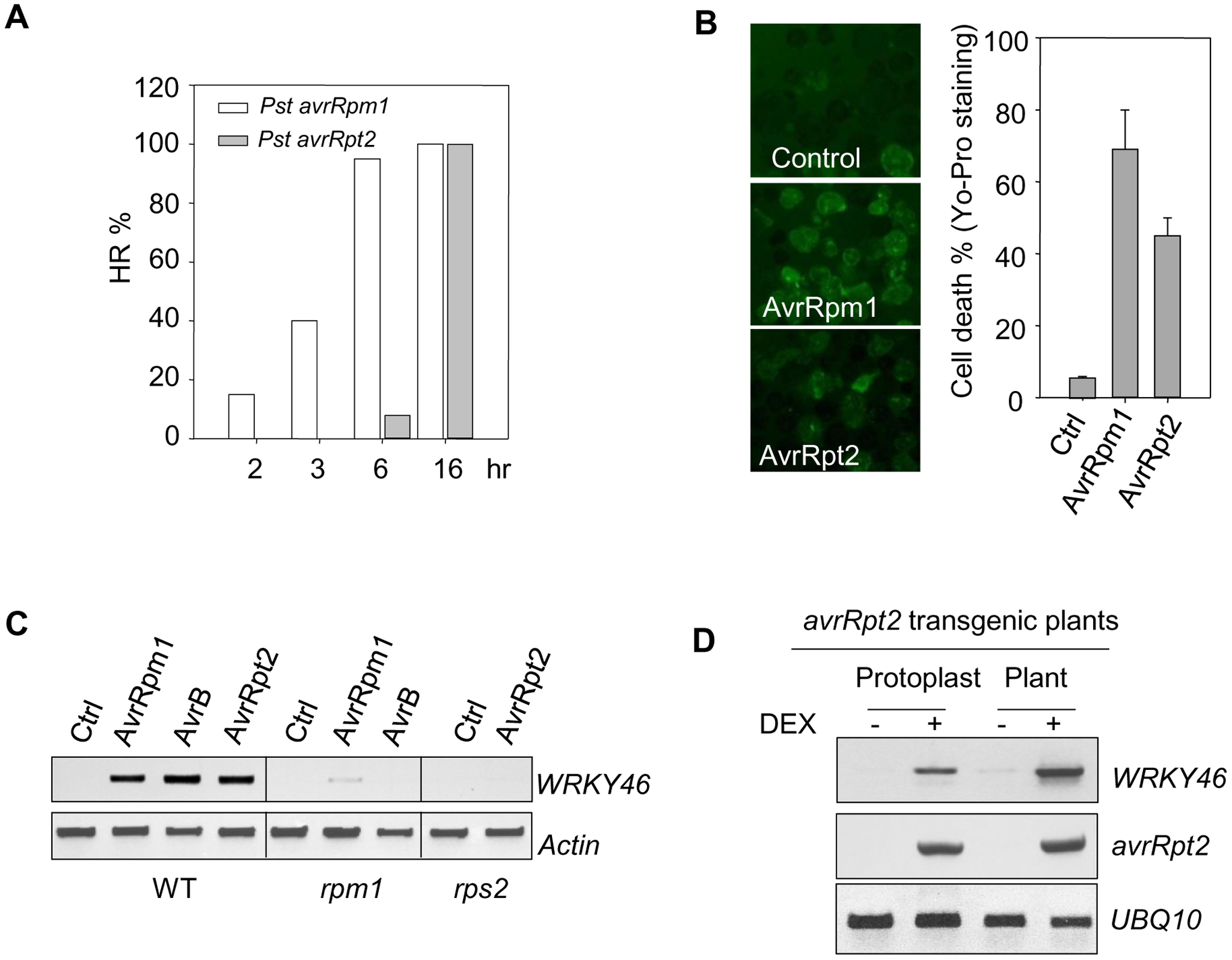

Supplement: Figure S1 — Effector induced cell death and gene activation in protoplasts and plants. (A) Hypersensitive response (HR)-induced by Pst avrRpm1 and avrRpt2 in plants. Arabidopsis leaves were inoculated with bacteria at 1×10^8^ cfu/ml. HR was indicated with the percentage of wilting leaves of total inoculated leaves (n>20) at the different time points after inoculation. Pst inoculation was used as a control. (B) Effector-induced cell death and nuclear fragmentation detected by YO-PRO-1 iodine staining at 16 hpt in protoplasts. (C) AvrRpm1, AvrB and AvrRpt2 activated endogenous WRKY46 expression in protoplasts. The transfected protoplasts were collected 3 hpt for RT-PCR analysis. The expression of Actin was used as a control. (D) Induction of WRKY46 expression in dexamethasone (DEX)-inducible avrRpt2 transgenic plants and protoplasts. The WRKY46 expression was detected 3 hr after DEX treatment. (TIF) [file ppat.1003127.s001.tif]

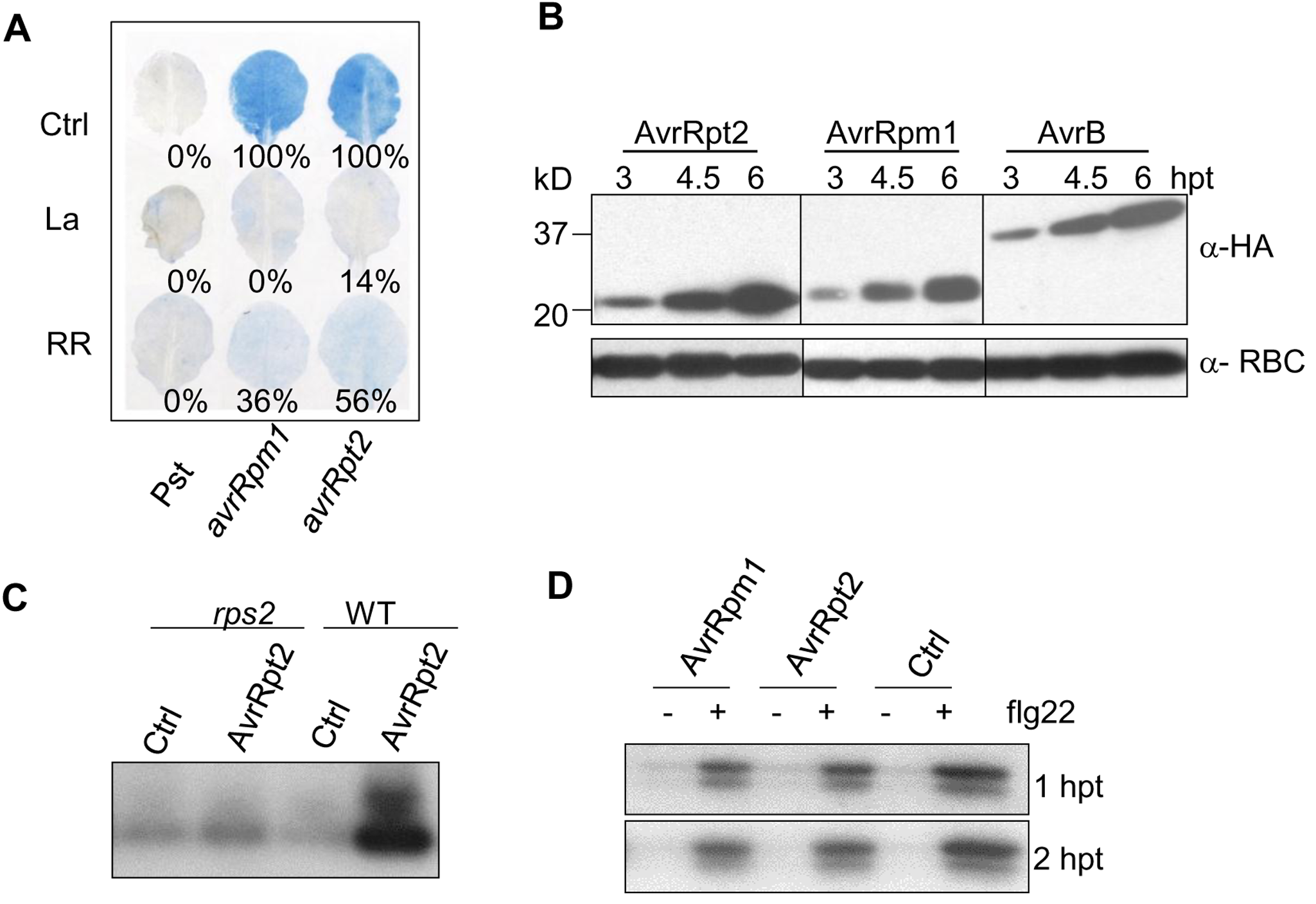

Supplement: Figure S2 — Ca2+ signaling in effector-triggered immunity. (A) Pst avrRpm1 and avrRpt2-induced cell death was suppressed by LaCl3 or RR treatment in plants. Arabidopsis leaves were inoculated with bacteria at 1×10^8^ cfu/ml in the presence of 2 mM LaCl3 or 20 µM RR. The cell death was shown by Trypan blue staining and % indicates the percentage of wilting leaves of total inoculated leaves (n>20). (B) Expression of effectors in Arabidopsis protoplasts. HA epitope tagged AvrRpt2, AvrRpm1 or AvrB was transfected in protoplasts and cells were collected at the indicated time for Western blot. To avoid cell death, AvrRpt2 was expressed in rps2, and AvrRpm1 and AvrB were expressed in rpm1 mutant protoplasts. (C) AvrRpt2-mediated CPK activation depended on RPS2 in protoplasts. The in-gel kinase assay using histone type III-S as substrate was performed 3 hpt. (D) Differential activation of MAPKs by flagellin and effectors in protoplasts. Ctrl, avrRpm1, or avrRpt2-transfected cells were incubated for 1 or 2 hr before the treatment with 1 µM flg22 for 10 min and subjected for an in-gel kinase assay using MBP as substrate. (TIF) [file ppat.1003127.s002.tif]

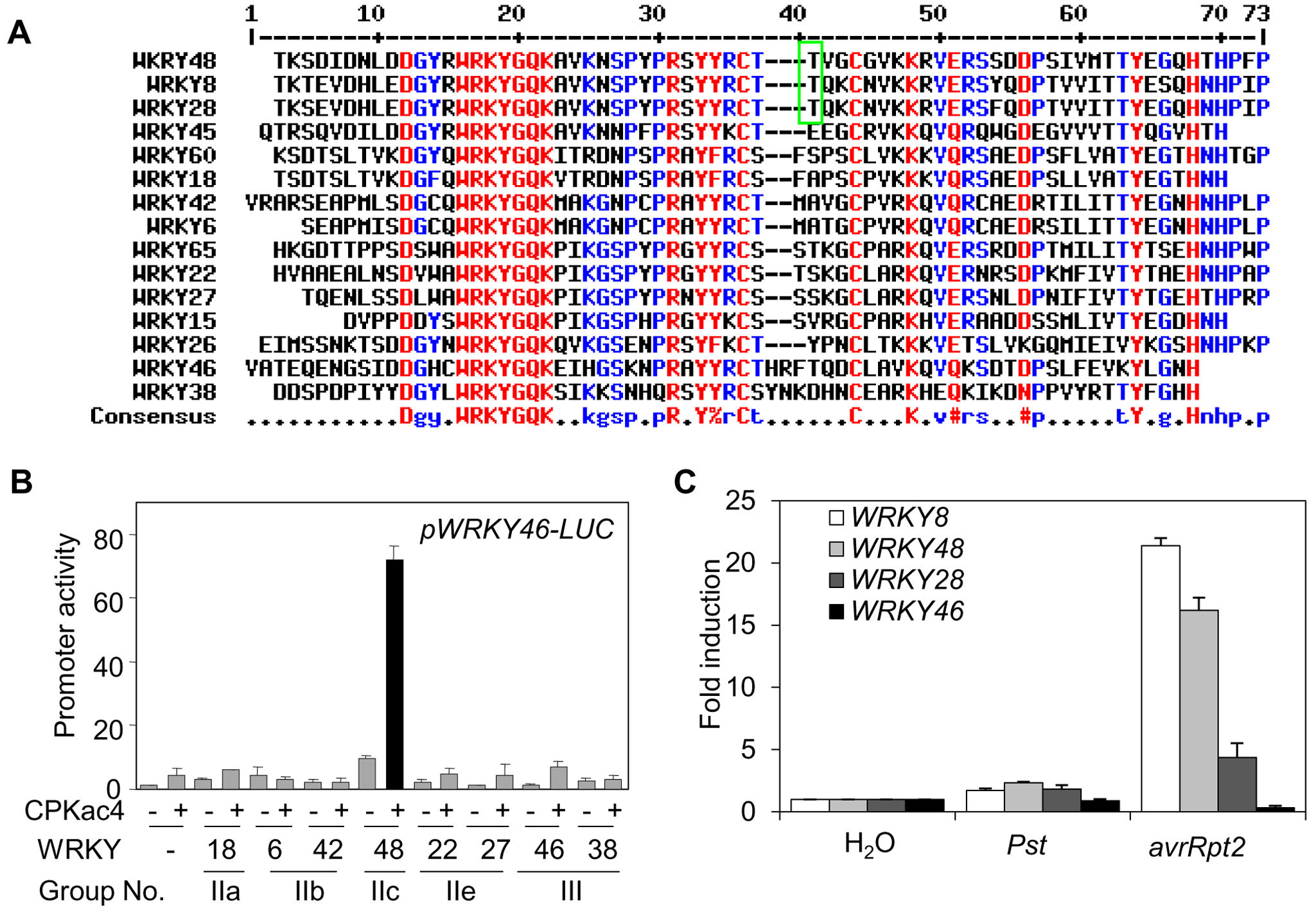

Supplement: Figure S3 — CPK and WRKY on WRKY46 promoter activity. (A) Alignment of DNA binding domains of WRKYs used in this study. The green box indicates the conserved Threonine (T) residue in WRKY48, 8 and 28. (B) Synergism of CPK4 and WRKYs on WRKY46 promoter activity in protoplasts. The representative WRKYs from different groups were co-transfected with CPKac4 for the activation of WRKY46 promoter. (C) Induction of WRKY8, 48, 28 and 46 by Pst and Pst avrRpt2 at 2 hpi in plants. Plant leaves were hand-inoculated with control or bacteria at 2×10^7^ cfu/ml. The samples were collected 2 hpi for real-time RT-PCR analysis. The expression of WRKY8, 48, 28 and 46 was normalized to the expression of UBQ10. The data are shown as the mean ± SE from three repeats. (TIF) [file ppat.1003127.s003.tif]

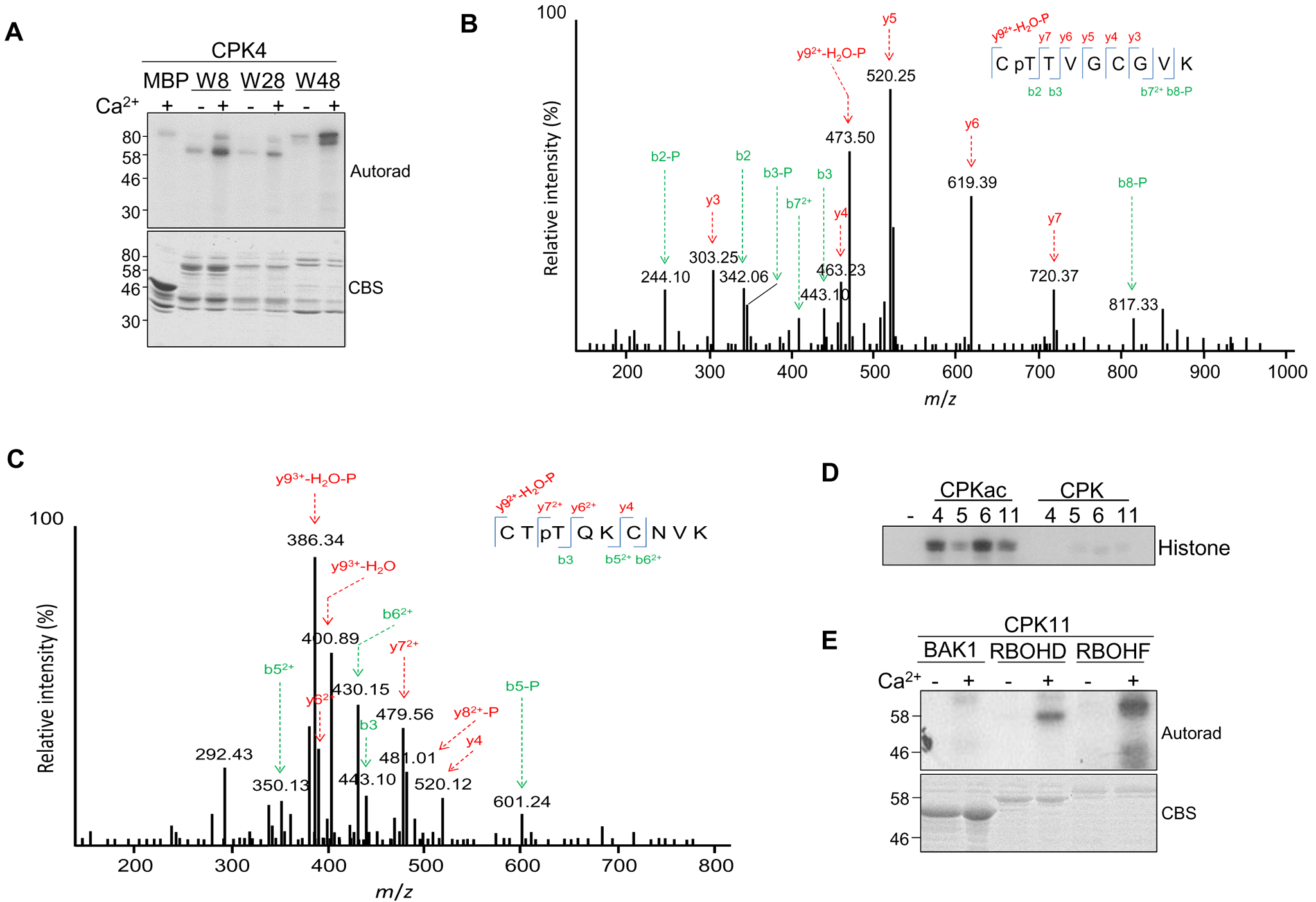

Supplement: Figure S4 — Phosphorylation of WRKY and RBOH by CPKs. (A) Phosphorylation of WRKYs by CPK4 in vitro. The recombinant MBP fusion proteins of WRKY8, 28 and 48 were used as the substrates for GST-CPK4 in an in vitro kinase assay in the presence of 1 mM Ca2+. (B) MS analysis identified WRKY48 T247 as a phosphorylation site by CPKs. Sequencing of a doubly charged peptide ion at m/z 531.21 that matches to CpTTVGCGVK of WRKY48. The confident b2 and b3 ions as well as y7 ion provide strong evidence for phosphorylation of the second Thr residue. (C) MS analysis identified WRKY28 T199 as a phosphorylation site by CPK5. Sequencing of a triply charged peptide ion at m/z 406.84 that matches to CTpTQKCNVK of W28. The confident b3 ion as well as y72+ ion provide strong evidence for phosphorylation of the third Thr residue. (D) Phosphorylation activity of CPKacs and CPKs on histone type III-S in vitro. FLAG-tagged CPKacs or WT CPKs were expressed in protoplasts and immunoprecipitated with α-FLAG antibody. The kinase activity was determined by in vitro assay using histone as a substrate. (E) Phosphorylation of RBOHD and RBOHF by CPK11 in vitro. The in vitro kinase assay was conducted in the presence of 1 mM Ca2+. BAK1, the kinase domain of receptor kinase BAK1, was used to show phosphorylation specificity. (TIF) [file ppat.1003127.s004.tif]

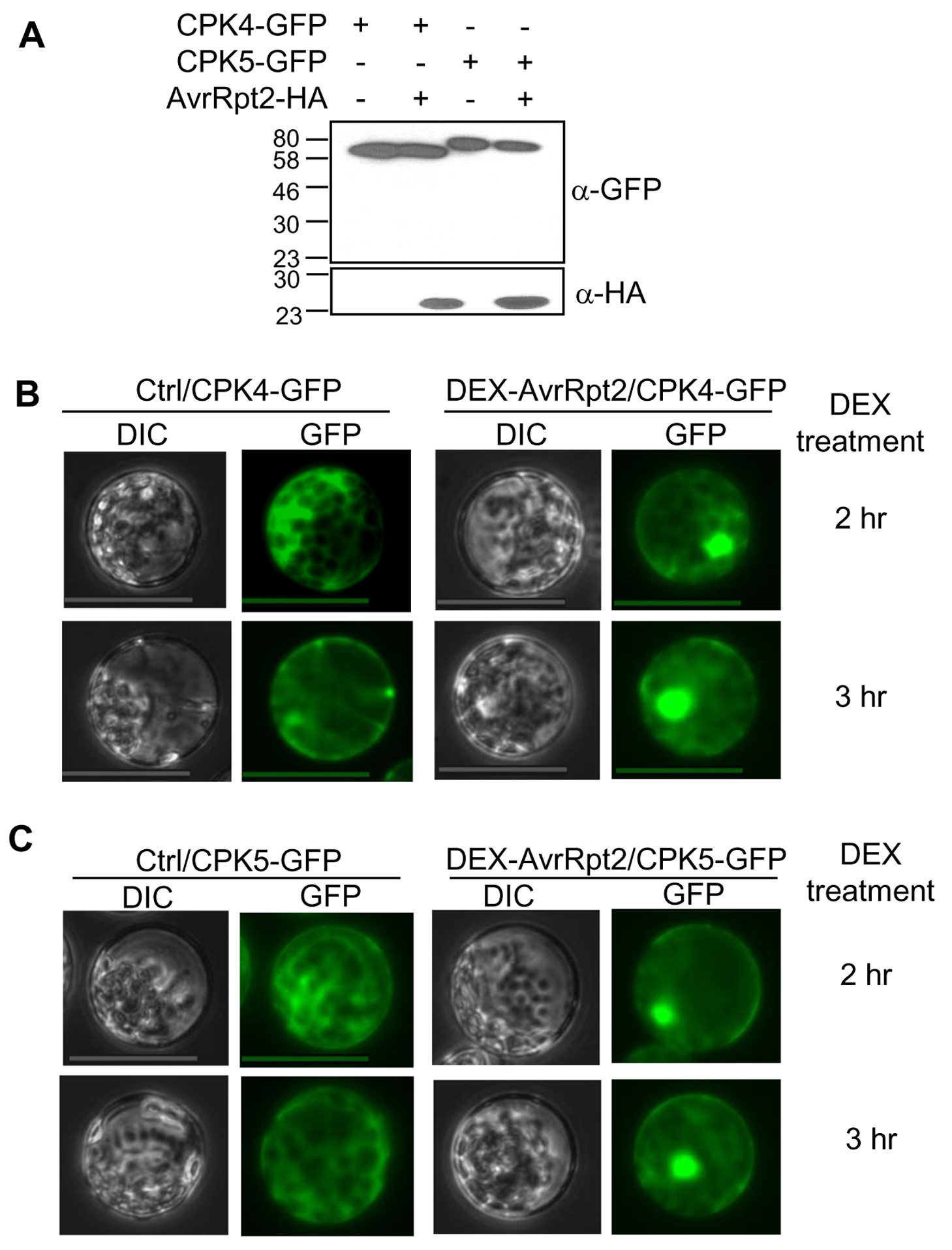

Supplement: Figure S5 — Effector AvrRpt2 stimulates CPK nuclear localization. (A) Expression of CPK4-GFP and CPK5-GFP in the presence of AvrRpt2-HA in protoplasts. Protoplasts were co-transfected with CPK4-GFP or CPK5-GFP and a vector control or AvrRpt2-HA, and expressed for 12 hrs. CPK expression was detected by Western blot with an α-GFP antibody, and AvrRpt2 expression was detected by an α-HA antibody. (B) AvrRpt2 stimulates CPK4-GFP nuclear localization in protoplasts. Protoplasts were co-transfected with CPK4-GFP and a vector control (Ctrl) or pTA7001-DEX-AvrRpt2. After expression for 10 hrs, the cells were treated with 10 µM of DEX for 2 or 3 hrs prior to observation of GFP localization. Bar = 50 µm. (C) AvrRpt2 stimulates CPK5-GFP nuclear localization in protoplasts. (TIF) [file ppat.1003127.s005.tif]

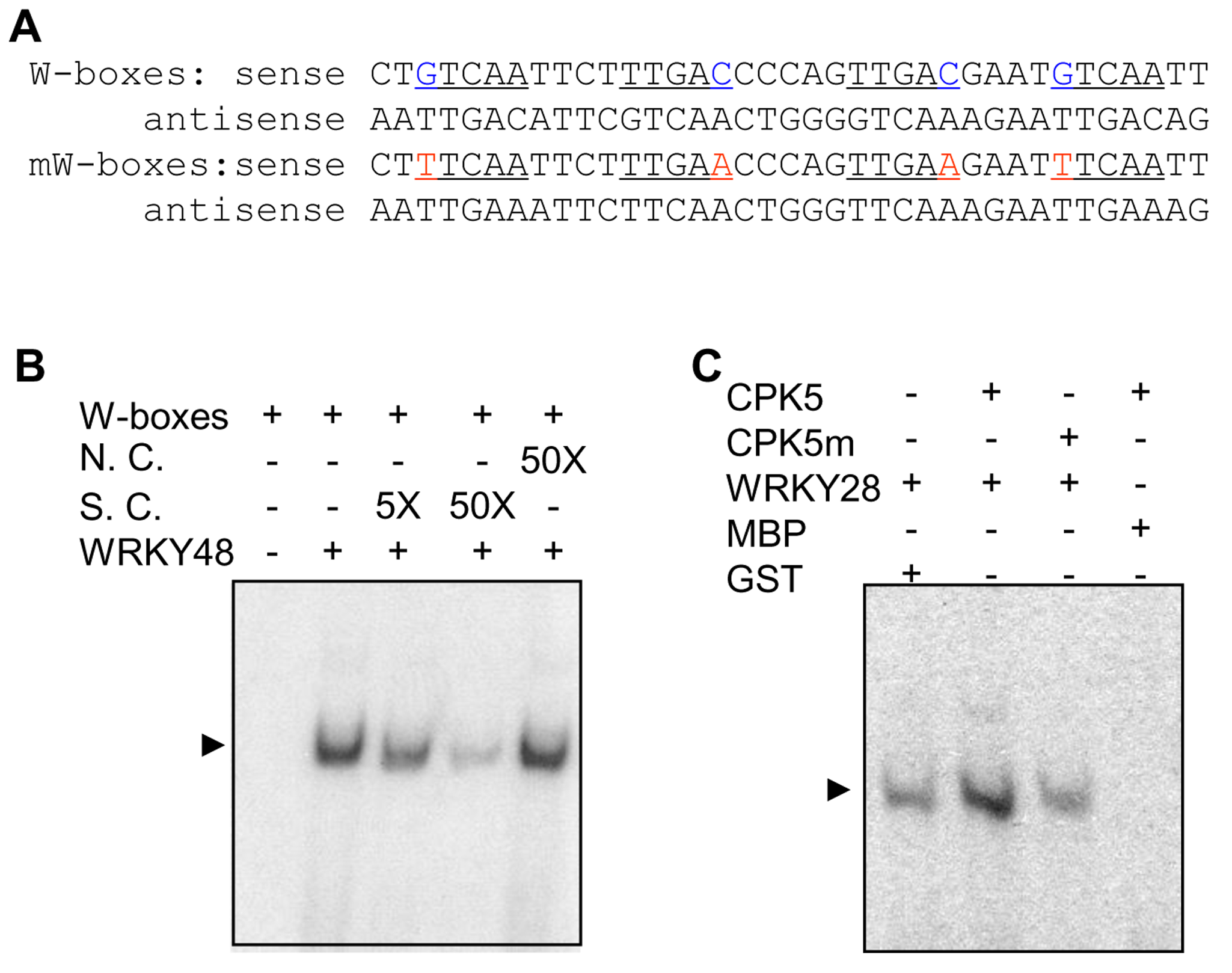

Supplement: Figure S6 — Specificity of WRKYs binding to the W-boxes. (A) Sequences of WT W-boxes probe and mutant W-boxes probe (mW-boxes). The W-box sequences corresponding to the WRKY46 promoter are underlined, and nucleotides in WT probe in blue were mutated in the mutant probe and colored in red. (B) Specificity of WRKY48 binding to the W-boxes in vitro. The recombinant WRKY48 protein was incubated with 32P-labeled W-boxes in a gel mobility shift assay. Specific competitor (S. C.) was non-labeled W-boxes oligonucleotide. Non-specific competitor (N.C.) was a random oligonucleotide. (C) Kinase activity is required for CPK-enhanced WRKY28 binding to the W-boxes in vitro. CPK phosphorylation of WRKY28 was conducted prior to DNA binding assay. (TIF) [file ppat.1003127.s006.tif]

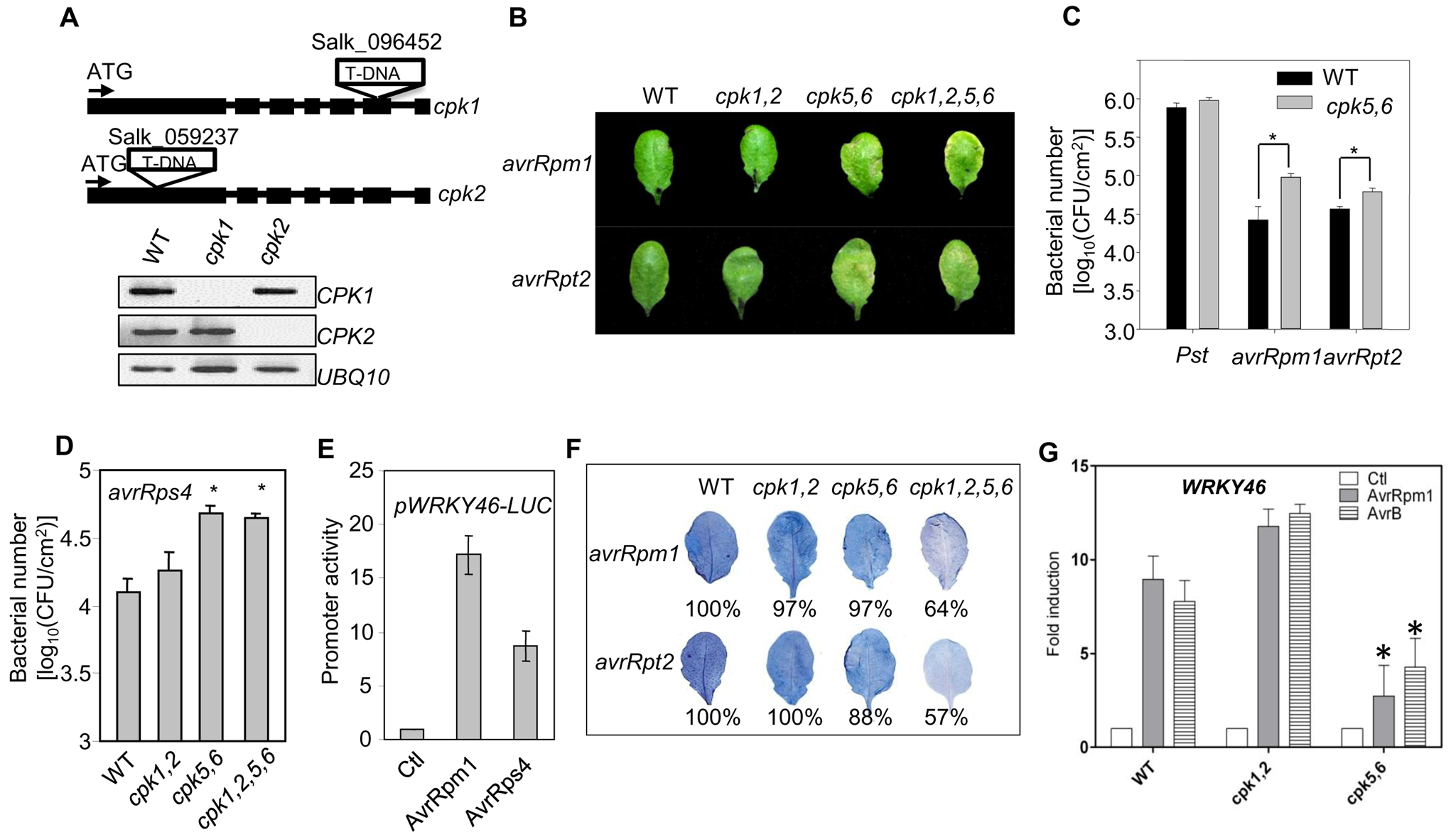

Supplement: Figure S7 — Analysis of cpk mutants. (A) T-DNA insertion sites and RT-PCR analysis in cpk1 and cpk2 mutants. (B) The disease phenotype of WT and cpk mutant plant by Pst avrRpm1 or avrRpt2 infection. Plant leaves were hand-inoculated with bacteria at 5×10^5^ cfu/ml. The picture was taken at 5 dpi. (C) The cpk5,6 mutant plants were compromised in avrRpm1- and avrRpt2-mediated disease resistance. Plant leaves were hand-inoculated with Pst, Pst avrRpm1 or Pst avrRpt2 at 5×10^5^ cfu/ml. The bacterial growth was measured 2 dpi. The data are shown as mean ± SE of three repeats, and the asterisk (*) indicates a significant difference with p<0.05 when compared with data from WT plants. (D) The cpk5,6 mutant plants were compromised in avrRps4-mediated disease resistance. Plant leaves were hand-inoculated with Pst avrRps4 at 5×10^5^ cfu/ml. The bacterial growth was measured 3 dpi. The data are shown as mean ± SE of three repeats, and the asterisk (*) indicates a significant difference with p<0.05 when compared with data from WT plants. (E) AvrRps4 activated WRKY46 promoter in protoplasts. The pWRKY46-LUC was co-transfected with AvrRpm1, AvrRps4 or a vector control in protoplasts and samples were collected at 6 hpt. The UBQ-GUS was included as an internal transfection control. The relative luciferase activity was normalized with GUS activity. (F) The cpk1,2,5,6 mutant plants diminished effector-mediated cell death. Plant leaves were hand-inoculated with Pst avrRpm1 or avrRpt2 at 1×10^8^ cfu/ml. The cell death ratio was recorded for avrRpm1 at 8 hpi and avrRpt2 at 16 hpi. The leaves were further stained with trypan blue to detect cell death. (G) Effector-induced WRKY46 expression was reduced in cpk mutant protoplasts. WRKY46 expression was detected in protoplasts 3 hpt by real-time RT-PCR analysis. The expression of WRKY46 was normalized to the expression of UBQ10. The data are shown as the mean ± SE from three independent biological replicates. (TIF) [file ppat.1003127.s007.tif]

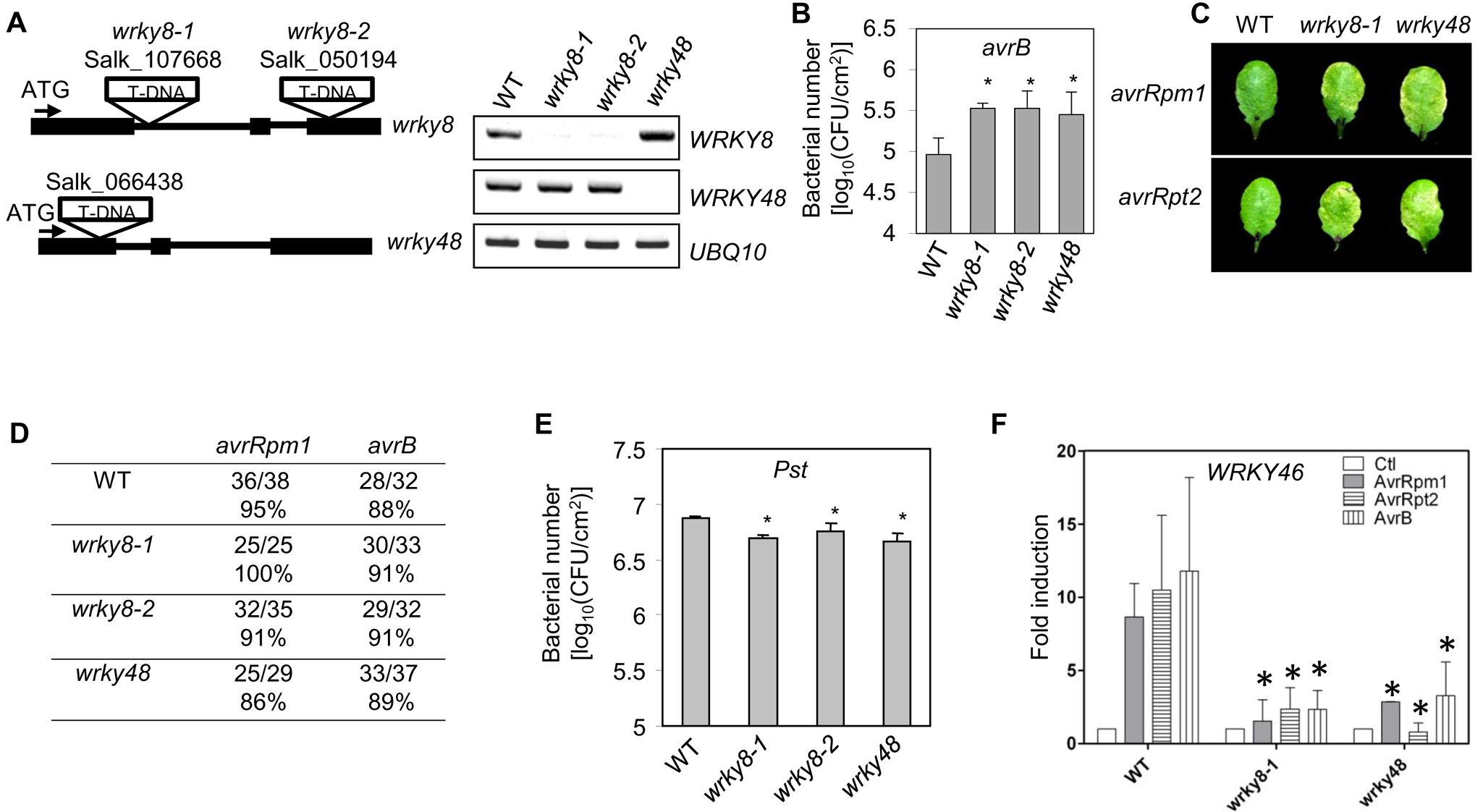

Supplement: Figure S8 — Analysis of wrky mutants. (A) T-DNA insertion sites and RT-PCR analysis in wrky8 and wrky48 mutants. (B) The bacterial growth of Pst avrB in wrky mutant plants. Plant leaves were hand-inoculated with Pst avrB at 5×10^5^ cfu/ml. The bacterial growth was measured at 3 dpi. The data are shown as mean ± SE of three repeats, and the asterisk (*) indicates a significant difference with p<0.05 when compared with data from WT plants. (C) The disease phenotype of WT and wrky mutant plants by Pst avrRpm1 or avrRpt2 infection. Plant leaves were hand-inoculated with different bacteria at 5×10^5^ cfu/ml and the pictures were taken at 6 dpi. (D) The cell death of wrky mutant plants. Plant leaves were hand-inoculated with Pst avrRpm1 or avrB at 1×10^8^ cfu/ml. The cell death ratio was recorded at 10 hpi, and indicated with the percentage (%) of wilting leaves of total inoculated leaves. (E) The wrky mutant plants are resistant to Pst infection. Plant leaves were hand-inoculated with Pst at 5×10^5^ cfu/ml. The bacterial growth was measured at 3 dpi. The data are shown as mean ± SE of three repeats, and the asterisk (*) indicates a significant difference with p<0.05 when compared with data from WT plants. (F) Effector-induced WRKY46 expression was reduced in wrky mutant protoplasts. WRKY46 expression was detected in protoplasts 3 hpt by real-time RT-PCR analysis. The expression of WRKY46 was normalized to the expression of UBQ10. The data are shown as the mean ± SE from three independent biological replicates. (TIF) [file ppat.1003127.s008.tif]
