# Supplementary material for: Bifurcation of Arabidopsis NLR Immune Signaling via Ca2+-Dependent Protein Kinases
Source: PLoS Pathog. 2013 Jan 31;9(1):e1003127. doi: 10.1371/journal.ppat.1003127 (PMC3561149; doi:10.1371/journal.ppat.1003127)
Supplement: Table S1 — Primers used in this study. (DOC) [file ppat.1003127.s009.doc]

**Table S1. Primers used in this study**

1) Cloning primers

| Gene | Forward primer | Reverse primer |
| --- | --- | --- |
| WRKY6 | CGGGATCCATGGACAGAGGATGGTCTGG | GAAGGCCTTTGATTTTTGTTGTTTCCTTCGCC |
| WRKY8 | CGGGATCCATGTCTCATGAAATCAAAGATC | AAGGCCTAGGCTCTTGTTTGAAGAAAACC |
| WRKY18 | CATGCCATGGACGGTTCTTCGTTTCTC | GAAGGCCTTGTTCTAGATTGCTCCATTAACC |
| WRKY28 | CGGGATCCATGTCTAATGAAACCAGAGATC | AAGGCCTAGGCTCTTGCTTAAAGAAAATTG |
| WRKY38 | CATGCCATGGAAATGAACTCCCCACACG | GAAGGCCTAAAGTAAAACTGATCATAACGATC |
| WRKY48 | CGGGATCCATGGAGAAGAAAAAAGAAGAGG | AAGGCCTTTTCTTATTCTCTTCATTGGTTTG |
| WRKY46 | CATGCCATGGTGATGGAAGAGAAACTTGTGATC | GAAGGCCTCGACCACAACCAATCCTG |
| WRKY46 promoter | CGGGATCCGCAGATGATAAGACATCATTTG | CATGCCATGGtcacttcagaaaattaagaatc |
| W28WRKY  domain | CGGGATCCATGGATCATCTTGAAGATGGT | GAAGGCCTGTGGTTGTGTTGACCCTC |
| W8WRKY  domain | CGGGATCCATGGATCATCTCGAAGACGGC | GAAGGCCTATGGTTGTGTTGACTCTC |
| W48WRKYdomain | CGGGATCCATGGATAATCTTGACGACGGTT | GAAGGCCTATGGGTATGCTGACCTTC |
| AtRbohD | CGGGATCCATGAAAATGAGACGAGGCAA | GAAGGCCTGAAGTTCTCTTTGTGGAAG |
| AtRbohF | CGGGATCCATGAAACCGT TCTCAAAGAA | GAAGGCCTGAAATGCTCCTTGTGAAATTC |

Note: restriction enzyme sites are underlined.

2) Point mutation primers

| Gene | Forward primer | Reverse primer |
| --- | --- | --- |
| pW46-LUC  W3 | ATTTCGATCTTTGGACCCCTAAAAATA | TATTTTTAGGGGTCCAAAGATCGAAAT |
| pW46-LUC  W1 | CTAATAGTCTCCTTTATTTCAATTTCTCTCC | GGAGAGAAATTGAAATAAAGGAGACTATTAG |
| pW46-LUC  W2 | GGGGAGTTAGTTGAAGATTTATAGATAAC | GTTATCTATAAATCTTCAACTAACTCCCC |
| pW46-LUC  W4 | GATAATACGTCACTTTCAATTGTGTGAATG | CATTCACACAATTGAAAGTGACGTATTATC |
| W48T247A | GCTATTACCGTTGCGCCACAGTGGGTTGCGGAGTG | CACTCCGCAACCCACTGTGGCGCAACGGTAATAGC |
| W48T248A | GCTATTACCGTTGCACCGCAGTGGGTTGCGGAGTG | CACTCCGCAACCCACTGCGGTGCAACGGTAATAGC |
| AtRbohD-S133A | CTTCCGAAGCACCGCCTCACGCATCAAG | CTTGATGCGTGAGGCGGTGCTTCGGAAG |
| AtRbohD-S148A | CCGCCGCGTGTTCGCTAGACGTCCCTCCC | GGGAGGGACGTCTAGCGAACACGCGGCGG |

Note: the nucleotides where point mutations occur are underlined.

3) Knockout primers

| Gene | Forward primer | Reverse primer |
| --- | --- | --- |
| *cpk1(Salk_096452)* | GTGATCTTGACTTTTCGTCCG | TTCTATGCGGACATCCTCAAC |
| *cpk2(Salk_059237)* | GTGACAAGCCTCTAAAACCCC | TTAAAATCATTGCCTGATCCG |
| *wrky8-1(Salk_*  *107668)* | TTGACTGCTTTTTGGCCATAC | CTCGATCAAGAGAACGGTTTG |
| *wrky8-2(Salk_*  *050194)* | CAAAAACAGTCCTTATCCGAGG | TTGTTTGAAGAAAACCGAAGG |
| *wrky48(Salk_066438)* | TTACCGGTGACCAGTGTTTTC | CTTTTTGGCCGTATTTTCTCC |

4) RT-PCR primers

| Gene | Forward primer | Reverse primer |
| --- | --- | --- |
| *CPK1* | GAAGCTGTTTCGGGAGAGCTT | TCCCCGTAGTCTTCTCGACA |
| *CPK2* | CGTTGGACCAAACATTTCTG | CTGGCTTGGTTTCAGACGTTG |
| *WRKY8* | GTTGACTCTCGTAGGTTGTGATG | TCCGAGGAGTTACTATAGATGCAC |
| *WRKY48* | AAGCCACAGTTGAAGGCAAA | TTTGGCCGTATTTTCTCCAC |
| *UBQ10* | AGATCCAGGACAAGGAAGGTATTC | CGCAGGACCAAGTGAAGAGTAG |
| *WRKY46* | CGTGCATCTGTAATATGCTCTAGG | GATGATGGTCACTGCTGGAG |
| *SID2* | CCCTTAACAAGGTTGTTCTTGC | CCTTCACGCTGTAACTGTGC |
| *PAD4* | AGATACGCGAGCACAACGCAAG | TTCTCGCCTCATCCAACCACTC |
| *EDS5* | AGTCTCATCGACACGGTCGTCATC | GCATAGTACTGTTCCCGGTCCAAG |
| *PBS3* | TCGCTGGCTTGTATAGGATGAGAG | ACTTAAACTGAGGCGCGTTGTTG |
| *PR1* | ACACGTGCAATGGAGTTTGTGG | TTGGCACATCCGAGTCTCACTG |
| *PR2* | AGCTTCCTTCTTCAACCACACAGC | TGGCAAGGTATCGCCTAGCATC |
| *PR5* | ATCACCCACAGCACAGAGACAC | AGCAATGCCGCTTGTGATGAAC |
| *WRKY30* | ttctcggagccaaatttccaagagg | ggcattgttgtaccgacatttgcag |
| *TUB4* | AGGGAAACGAAGACAGCAAG | GCTCGCTAATCCTACCTTTGG |

5) Primers for Chip assays

| A-WRKY46chipF | caagctaagagcagcccaaa |
| --- | --- |
| A-WRKY46chipR | gcgtctggtcaagggaataa |
| B-WRKY46chipF | attcagtacgatgaatatggcaaa |
| B-WRKY46chipR | gaaatttttaatcatggcgctta |
| C-WRKY46chipF | cgtcactgtcaattgtgtgaa |
| C-WRKY46chipR | agggggtcaaagatcgaaat |
| D-WRKY46chipF | tcacgtcagcgaaacaaaag |
| D-WRKY46chipR | aagtcggttaagctaatcctatttta |
| E-WRKY46chipF | tcagaaacgagtaacccctaga |
| E-WRKY46chipR | cgaccagaagttgtgtggaa |
| F-WRKY46chipF | AACATCACATCCCCGAAGAC |
| F-WRKY46chipR | TTTCCACACACCGTTCTCAA |
| A-SID2chipF | tttagccagaacggacttgg |
| A-SID2chipR | atcttaccttacgcggcaga |
| B-SID2chipF | tgtctcgagttcaacccttg |
| B-SID2chipR | aaacagagcataatggaaaacaca |
| C-SID2chipF | cacgctttgtcacacaaaaa |
| C-SID2chipR | ttgctttcacactgacgctaa |
| D-SID2chipF | gcaatgatgcttgttgaattatg |
| D-SID2chipF | actcaacctggtcaaaatgaa |
| E-SID2chipF | ctctttgccagtctcgtgtg |
| E-SID2chipR | tgctaggaaacccacttaggc |
| F-SID2chipF | agcatgcgtgtaatgccata |
| F-SID2chipR | gttcctctcgtgtgggtgtt |
| G-SID2chipF | aacacccacacgagaggaac |
| G-SID2chipR | cggattcgtttggttgattt |
| H-SID2chipF | ATTCTCCCGCAAGgtctttt |
| H-SID2chipR | GGCGTCTTGAAATCTCCATC |
| J-SID2chipF | ttttggctcgtggaattagG |
| J-SID2chipR | GATATGCATCATGCCCTTCA |
| K-SID2chipF | CCCATCTTCAGAGTGGAATGA |
| K-SID2chipR | AACCCCTTATCCCCCATACA |
| CAB1chipF | tcacgtcatgagtgggtgtt |
| CAB1chipR | tggtattgatgcgattttcg |
